# Genome editing and transcriptional repression in *Pseudomonas putida* KT2440 via the type II CRISPR system

**DOI:** 10.1186/s12934-018-0887-x

**Published:** 2018-03-13

**Authors:** Jun Sun, Qingzhuo Wang, Yu Jiang, Zhiqiang Wen, Lirong Yang, Jianping Wu, Sheng Yang

**Affiliations:** 1 Institute of Bioengineering, College of Chemical and Biological Engineering, Zhejiang University, Hangzhou, 310027 China; 2 Key Laboratory of Synthetic Biology, Institute of Plant Physiology and Ecology, Shanghai Institutes for Biological Sciences, Chinese Academy of Sciences, Shanghai, 200032 China; 3 Shanghai Research and Development Center of Industrial Biotechnology, Shanghai, 201206 China; 4 Jiangsu National Synergetic Innovation Center for Advanced Materials, SICAM, Nanjing, 210009 China

**Keywords:** CRISPR–Cas9 system, *Pseudomonas putida* KT2440, Genome editing, Single nucleotide mutation, Transcriptional engineering, CRISPR–Cpf1 system

## Abstract

**Background:**

The soil bacterium *Pseudomonas putida* KT2440 is a “generally recognized as safe”-certified strain with robust property and versatile metabolism. Thus, it is an ideal candidate for synthetic biology, biodegradation, and other biotechnology applications. The known genome editing approaches of *Pseudomonas* are suboptimal; thus, it is necessary to develop a high efficiency genome editing tool.

**Results:**

In this study, we established a fast and convenient CRISPR–Cas9 method in *P. putida* KT2440. Gene deletion, gene insertion and gene replacement could be achieved within 5 days, and the mutation efficiency reached > 70%. Single nucleotide replacement could be realized, overcoming the limitations of protospacer adjacent motif sequences. We also applied nuclease-deficient Cas9 binding at three locations upstream of enhanced green fluorescent protein (eGFP) for transcriptional inhibition, and the expression intensity of eGFP reduced to 28.5, 29.4, and 72.1% of the control level, respectively. Furthermore, based on this CRISPR–Cas9 system, we also constructed a CRISPR–Cpf1 system, which we validated for genome editing in *P. putida* KT2440.

**Conclusions:**

In this research, we established CRISPR based genome editing and regulation control systems in *P. putida* KT2440. These fast and efficient approaches will greatly facilitate the application of *P. putida* KT2440.
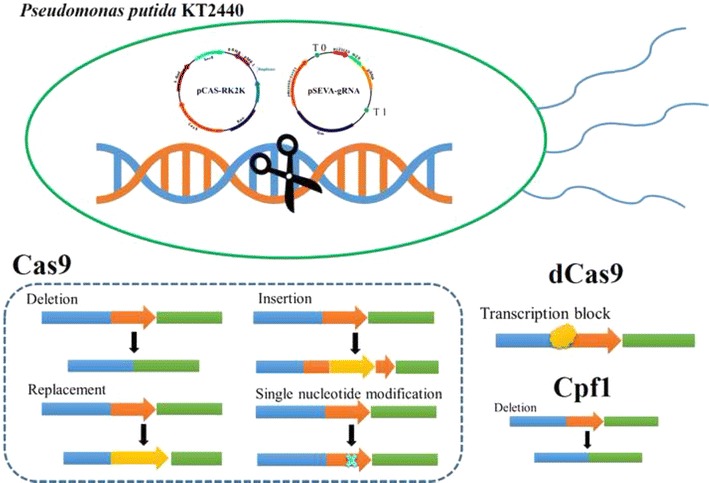

**Electronic supplementary material:**

The online version of this article (10.1186/s12934-018-0887-x) contains supplementary material, which is available to authorized users.

## Background

*Pseudomonas putida* KT2440 is an environmentally safe, non-pathogenic Pseudomonad species [1]. Because of its outstanding capacity to degrade aromatics compounds and robust viability in harsh conditions, *P. putida* KT2440 is an ideal chassis for bioremediation [2], metabolic engineering [3], and biocatalysis [4]. Several genome editing approaches have been applied into *Pseudomonas*. A series of molecular tools, such as counter-selection markers (*sacB* [5], *pyrF* [6], *upp* [7]), heterologous recombinases (Cre-*loxP* [8], Flp-*FRT* [9]), and suicide vectors [10] have been validated and spurred the advancement of allelic exchange for engineering mutations in this genus. Bacteriophage-based recombination proteins (Red/ET [11], λ-Red [12, 13]) enable homologous recombination between a target genome locus and donor DNA, which is an efficient method in *Pseudomonas aeruginosa* PAO1 [12] and *P. putida* KT2440 [11, 13]. Based on homologous recombination in double-stranded breaks (DSB), the I-SceI homing endonuclease has been developed as a seamless genome editing tool in *P. putida* KT2440 [14]. In another chromosomal engineering approach used in *Pseudomonas*, transposon vectors-based methods [15–17] have excellent transposition frequencies. Although these various strategies have been applied into genome engineering of *Pseudomonas*, they still have many drawbacks, such as time-consuming manipulation, scars left in the genome, inability to target exact loci, difficulty in generating mutants, and deficiency in plasmid-curing.

Clustered Regularly Interspaced Short Palindromic Repeats (CRISPR)-Cas (RNA-guided proteins) systems are a prokaryotic adaptive immune defense mechanism in many bacteria and most archaea [18]. Among three major types, the type II CRISPR–Cas system from *Streptococcus pyogenes* (SpCas9) is the best characterized [19]. In the CRISPR–Cas9 system, a chimeric single guide RNA (sgRNA) is used for Cas9 sequence-specified guidance [20], and a short protospacer adjacent motif (PAM) exerts recognition between Cas9 and the target DNA [21]. Cas9 protein then catalyzes the breaking of DNA double strands [22]. The DSB caused by Cas9 can be repaired via homology-directed repair (HDR) [23] or non-homologous end joining (NHEJ) [24] in eukaryotes. Cas9-nickase (nCas9), in which one domain is inactivated (D10A or H840A variant), can reduce the lethal effect on cells and the repairing process can occur more easily [25, 26]. A nuclease-deficient Cas9 (dCas9) retains sgRNA binding ability and can be applied for target gene regulation, including blocked transcription for CRISPR-based interference [27], or fused with activators for RNA-guided activation [28]. Because of its marker-free, cost-efficient, simple genome manipulation, the CRISPR–Cas9 system has been developed as a genome engineering tool in a wide range of prokaryotes and eukaryotes, including (but not limited to) *Escherichia coli* [29], *Clostridium cellulolyticum* [30], *Streptomyces* species [31], *Bacillus subtilis* [32], *Yarrowia lipolytica* [33], *Saccharomyces cerevisiae* [34], mammalian cells [35], and zebrafish [36]. Recently, Aparicio et al. [37] developed a CRISPR/Cas9-based three-plasmid system in *P. putida*, and gene deletion or single nucleotide substitution can be achieved. However, this three-plasmid system has challenges regarding plasmid-curing, especially for continual genome editing, and other versatile characteristics of type II CRISPR system have not been fully explored in *P. putida* KT2440.

Cpf1 is a newly discovered single RNA-guided nuclease [38] that belongs to type V-A CRISPR systems [39]. In contrast with the chimeric sgRNA-associated Cas9, in the CRISPR–Cpf1 system, Cpf1 recognizes ‘TTN’ PAM sequence. The PAM sequence is located at the 5′- end of the target sequence, and the spacer sequence follows a 19-nt CRISPR RNA (crRNA) direct repeat. Aside from the different recognition mechanism compared with Cas9, the staggered cutting style of Cpf1 could facilitate gene insertion for NHEJ repair mechanisms [38]. Taken together, these characteristics make CRISPR–Cpf1 an attractive complementary tool to the well-established CRISPR–Cas9 system.

Because of the versatile functions and simple manipulation of CRISPR–Cas9, we developed a CRISPR–Cas9 based genome editing system to overcome major limitations of the known gene editing technologies of *P. putida* KT2440. Here, we tested Cas9 toxicity and analyzed sgRNA off-target effect; multiple genes were targeted for integration or deletion by our two-plasmid system. Catalytically inactive Cas9 (dCas9) was applied to control gene expression via transcriptional repression. Additionally, we preliminarily explored the CRISPR–Cpf1 system for genome editing in *P. putida* KT2440. These systems could be powerful tools to extend the application of *P. putida* KT2440, and pave the way for use of CRISPR systems into other Pseudomonads.

## Methods

### Strains, culture conditions and reagents

The strains and plasmids used in this study are given in Additional file 1. All kinds of *E. coli*, and *P. putida* KT2440 were grown in LB medium (Liquid and Solid). *E. coli* DH5α was used for all cloning and maintenance, while *E. coli* S17-1 served as a helper strain for conjugal transfer. *E. coli* were grown at 37 °C, and *P. putida* KT2440 was incubated at 30 °C. Antibiotics were added at the following concentrations: kanamycin, 150 μg/mL (50 μg/mL for *E. coli*); gentamicin, 50 μg/mL; tetracycline, 25 μg/mL (15 μg/mL for *E. coli*) and spectinomycin, 100 μg/mL.

The DNA polymerase PrimeSTAR^®^ Max, all restriction endonucleases and T4 DNA ligase were purchased from Takara Bio Inc (Dalian, China). The One Step Cloning Kit (Vazyme Biotech Co., Ltd, Nanjing, China) and pEASY-Uni Seamless Cloning and Assembly Kit (TransGen Biotech, Beijing, China) were applied for seamless cloning.

### Plasmids construction and N20 sequence selection

**Fig. 1:**
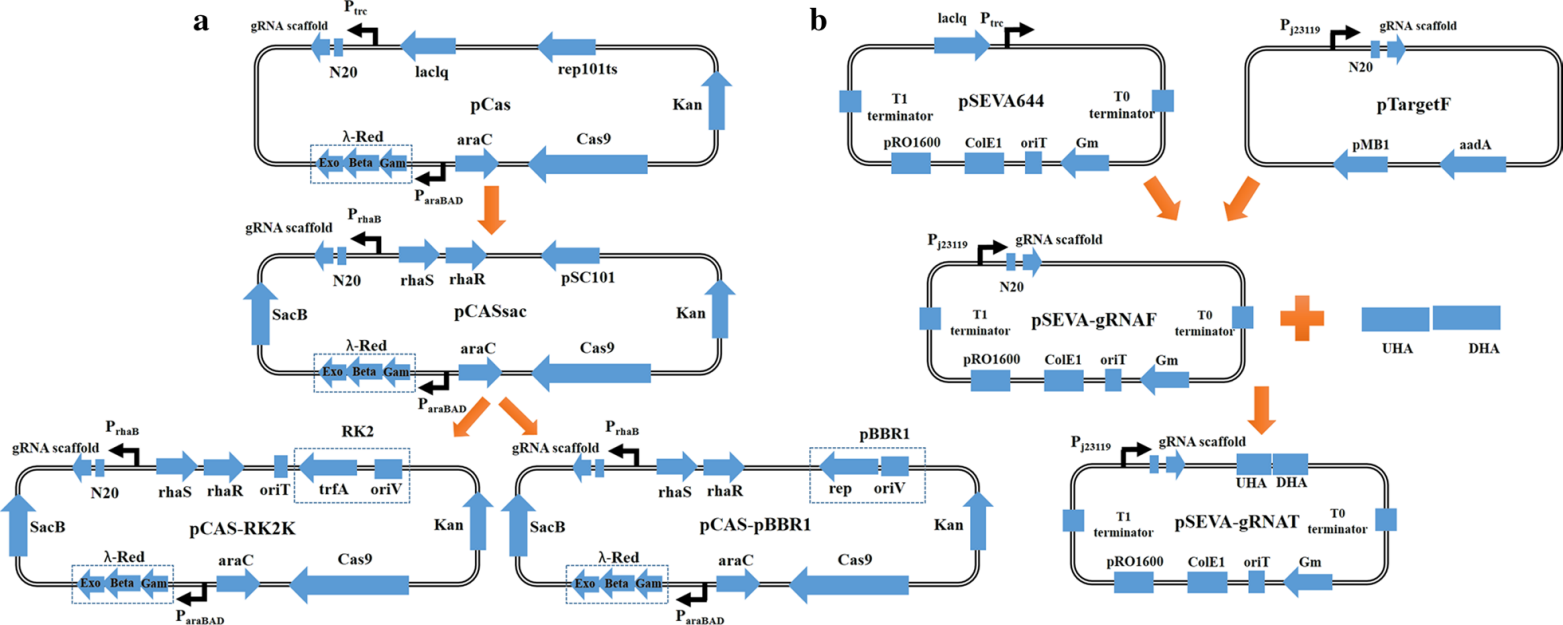
Strategy for the construction of a CRISPR–Cas9 two-plasmid system in *P. putida* KT2440. **a** *pSC101* replicon in pCASsac was replaced with *RK2* replicon together with *oriT* fragment, creating pCAS-RK2K. In pCAS-RK2K, *cas9* gene was linked with its native promoter, and gRNA cassettes were transcribed by *P*_*rhaB*_ promoter so as to guide *Cas9* protein targeting *pRO1600* replicon in pSEVA-gRNAT. The *λ*-*Red* recombination system was under control of arabinose promoter to enhance the repairing efficiency in KT2440. *SacB*, a commonly used counterselectable marker function as a self-curing tool. **b** The *P*_*trc*_-*lacl*^*q*^ inducible system was eliminated from pSEVA644 and gRNA cassettes were inserted, generating pSEVA-gRNAF. The upstream homologous arm (UHA) and downstream homologous arm (DHA) were amplified from genome and connected by overlap-extension PCR. Next, the combinational homologous arm was assembled into pSEVA-gRNAF, giving rise to pSEVA-gRNAT

The CRISPR/Cas9 system consists of two plasmids: pCAS-RK2K and pSEVA-gRNAT (Fig. 1). Plasmid pCAS-RK2K was constructed from plasmid pCASsac (an unpublished *E. coli* CRISPR/Cas9 2.0 system), which was a gift from Yang Sheng. Plasmid pSEVA-gRNAT was derived from pTargetF [29] and pSEVA644, the latter together with its same series of plasmids was obtained from SEVA Database [40]. All primers used in this study are listed in Additional file 2.

Based on pCAS backbone [29], the construction of pCASsac focused on the following steps: a tightly-controlled Rhas-PrhaB promoter replaced with lacIq-Ptrc promoter; pSC101 replicon as a substitute for the temperature-sensitive replicon repA101 (Ts); SacB acted as a curing component was inserted into the plasmid. In our study, two broad-host-range replicons, RK2 (together with oriT fragment) from pSEVA429 and pBBR1 from pBBR1MCS2 were amplified using primers Sa-RK-1F/Sa-RK-2R and Sa-BBR1-1F/Sa-BBR1-2R, respectively. The replication origin of pCASsac was then replaced with RK2 or pBBR1 using the One Step Cloning Kit (the detailed construction strategies are shown in Additional file 3), thus creating plasmids pCAS-RK2K and pCAS-pBBR1 (Fig. 1a), respectively. pCAS-RK2K has a tetracycline version: pCAS-RK2T (the kanamycin resistance marker in pCAS-RK2K was replaced with a tetracycline resistance marker). Elimination of Cas9 or λ-Red inducible expression system from pCAS-RK2K respectively, pCAS-RK2K△Cas9 and pCAS-RK2K△Red were constructed via Gibson assembly with primers QC-1F/QC-1R, XC-2F/XC-2R and JH-1F/JH-1R, JH-2F/JH-2R, JH-3F/JH-3R.

Plasmid pSEVA-gRNAT (Fig. 1b) was assembled from the following fragments: LacIq-ptrc promoter was removed from pSEVA644, and then pSEVA644△LacIq-ptrc was used as backbone; j23119 promoter and sgRNA cassette from pTargetF were fused into the *Eco*RI/*Sac*I digested pSEVA644△LacIq-ptrc via primers JS-1F/JS-2R. We selected *nicC* (Locus tag is PP_3944) as target site, which is an unessential gene in the *P. putida* KT2440 genome. pSEVA-gRicF was derived from pSEVA-gRNAT by replacing the original N20 sequence with ‘AAAATCGCAATCGTCGGTGC’ via inverse PCR using primers NC-F/NC-R. The homologous arms of *nicC* were acquired from the Pseudomonas Genome Database [41]. Next, 500 bp length upstream and downstream of the target gene were amplified using primers NT-1F/NT-2R, NT-ZF/NT-ZR, and connected via overlap-extension (SOE) PCR. The resulting homologous arm was then inserted into the *Bam*HI/*Hin*dIII-digested pSEVA-gRicF, thus creating pSEVA-gRicT.

Here, the N20 sequence of low off-target rate was designed via CasOT [42]. N20 sequences of all pSEVA-gRNAT derivatives were constructed by two reverse primers with 20 nt reverse complementarity. The repairing homologous arms were ligated into pSEVA-gRNAT by *Bam*HI and *Hind*III sites or using Gibson assembly. Toward different DNA template strand of *nicC* (PP_3944), pSEVA-gRic6T and pSEVA-gRic5T were constructed from pSEVA-gRicT via primers Nic6F/Nic6R and Nic5F/Nic5R, respectively. The target-specificity of pSEVA-gRic6T and pSEVA-gRic5T was calculated by CasOT. Due to its specificity in the genome, the N20 sequence (‘CATTCAGAACTAACTTGTCG’) was inserted into pSEVA-gRNAT via primers DgRNA-F/DgRNA-R, thus creating pSEVA-dgRNA. pSEVA-dgRNA was designed as a positive control. The sequence ‘GGTTGTAGGAAGATTCGATA’ from pSEVA-gRNAT replicon (pRO1600) was selected as N20 sequence under the transcriptional control of rhamnose-inducible promoter (Rha), in which pCAS-RK2K was applied to cure pSEVA-gRNAT. All of the N20 sequences designed by CasOT and primers used for pSEVA-gRNAT derivatives are listed in Additional files 2 and 4.

### Plasmid transformation method

Plasmid pCAS-pBBR1, pCAS-RK2K, pCAS-RK2T, pSEVA-gRNAT or other derivatives was transformed into *P. putida* KT2440 by electroporation. Firstly, KT2440 was inoculated and cultivated overnight, then transferred to a 100 mL flask containing 10 mL LB without antibiotics until cell densities (OD_600_) reached 0.6–0.8. After concentration, the electrocompetent cells were prepared by three times washing of 3 mM HEPES Buffer [13], then concentrated to 500 µL. For electroporation conditions, 100 µL bacterial suspension and 150 ng DNA were transferred to a 2 mm gap ice-cold electroporation cup and the detailed setting of Bio-Rad GenePulser II were listed as follows: 2.5 kV; 200 V; 25 uF. After electroporation, 1 mL LB liquid medium was added and mixed, then the entire mixture was transferred into a 2.0 mL Eppendorf tube and cultivated in 30 °C for 2 h before being spread on LB selection plates.

Alternatively, *E. coli* S17-1 could be used as a helper strain to transfer pCAS-RK2K or its derivatives into *P. putida* KT2440. *E. coli* S17-1 containing plasmid (adding related antibiotics), and *Pseudomonas* species were cultivated in LB medium overnight, then each bacteria was transferred to a 5 mL LB tube (without antibiotics) for incubation until the OD_600_ reached 0.6–0.8. All these cells were concentrated and washed four times with sterile water, then *E. coli* mixed with *Pseudomonas* in a ratio of 1:1. Next, these mixtures were dripped on LB agar plates in small dots, and incubated at 37 °C for 6 h, then shifted to 30 °C overnight. After incubation, the mixture cells were scraped from the plate, and diluted and spread on selection plates (Tetracycline 25 μg/mL or kanamycin 150 μg/mL, together with spectinomycin 100 μg/mL) for 12–16 h at 30 °C.

### Toxicity analysis

As the catalytic activity of Cas9 influences its toxicity in several prokaryotic microbes, we constructed four versions of Cas9 by introducing point mutations at the D10A, H840A, D10A and H840A together, as well as frameshift mutation in the start codon by transforming ATG to AGTG (Cas9FM) via primers PDF1F/PDF1R, PDF2F/PDF2R, and PDFM-1F/PDFM-1R, respectively. Each Cas9 derivative was assembled into pCAS-RK2K△Cas9, creating the following plasmid pCAS-RK2K-nCas9D, pCAS-RK2K-nCas9H, pCAS-RK2K-dCas9, and pCAS-RK2K-Cas9FM. In this experiment, pCAS-RK2K was used as a control. Using the same electroporation conditions, 150 ng of these plasmids were transformed into *P. putida* KT2440 in respective triplicate, and then screened by kanamycin plates. After cultivated at 30 °C for 18 h, the average number of transformants was calculated as CFU/μg of DNA.

### Genome editing

In this two-plasmid system, large size plasmid pCAS-RK2K or pCAS-RK2T was first transformed into *P. putida* KT2440 by electroporation or conjugal transfer.

After preparation of KT2440 harboring pCAS-RK2K or pCAS-RK2T, candidate colony was inoculated in LB liquid medium and cultivated overnight. Then overnight culture was transferred into 10 mL tube by 4%, and 0.6% l-arabinose (0.06 g in 10 mL LB liquid) was added into the medium and completed cultivation about 2–2.5 h. The bacteria cells were then harvested and washed by 3 mM Hepes Buffer [13], and finally concentrated to 500 μL for electroporation. Using the electroporation protocols described above, 150 ng of pSEVA-gRNAT or its derivatives was added. After the recovery process, 100 μL final concentrated cells were plated on selection plates (Tetracycline 25 μg/mL or kanamycin 150 μg/mL, together with gentamycin 50 μg/mL) and cultivated at 30 °C. Transformants were analyzed by colony PCR and confirmed by DNA sequencing.

In order to investigate the deficiency effect of four essential sections (Cas9, gRNA cassette, homologous repairing template, and λ-Red system) in this system, we designed a series of control experiment to calculate the total CFU and analysed the mutation efficiency. Plasmid pCAS-RK2K, pCAS-RK2K△Cas9 and pCAS-RK2K△Red (plasmid construction see above) were first transformed into *P. putida* KT2440, respectively. After KT2440 harboring the related pCAS-RK2K or its derivatives, pSEVA644, pSEVA-dgRNA, pSEVA-gRic6T, and pSEVA-gRic6F (derived from pSEVA-gRic6T by deletion of homologous repairing template) were sequential transformed into these strains, respectively (see below Fig. 3a).

### Plasmid curing

After the identification procedure, the mutated colony was inoculated into a 5 mL LB medium with kanamycin (150 μg/mL) or tetracycline (25 μg/mL) plus rhamnose (10 mM) and shaken at 30 °C overnight. Subsequently, the colonies were streak on LB selection plates containing kanamycin (150 μg/mL) or tetracycline (25 μg/mL), and confirmed as pSEVA-gRNAT lost by colony PCR via primers PS1/PS2. After the first round of genome editing, the pSEVA-gRNAT cured strain could be used in the next round of genome editing. Finally, a plasmid-cured colony was obtained by cultivated overnight with glucose (5 g/L), then diluted and streak in LB agar plates adding sucrose (10 g/L) and glucose (5 g/L). The curing of pCAS-RK2K was confirmed by colony PCR via primers Ra-JF/Ra-JR or identified its antibiotics sensitivity.

### Single nucleotide mutation

The construction of single nucleotide mutation in the genome can be divided into two strategies.

In the first strategy, single nucleotide mutation aimed to change the PAM sequence (a 3-nt upstream of N20 sequence for pSEVA-gRic6T), the nucleotide sequence ‘CGG’ to ‘CAG’ within *nicC* gene. We amplified the first 500 bp of *nicC* and its upstream 500 bp length sequence from genome by primers NCD1F/NCD2R. Based on pSEVA-gRic6T backbone, we assembled the fragment into the plasmid (creating pSEVA-gRic6PAM1) via Gibson assembly and then site-directed mutation in ‘CGG’ was performed by primers NPAM-F/NPAM-R, generating pSEVA-gRic6PAM2. After electroporation of pSEVA-gRic6PAM2 into KT2440 harboring pCAS-RK2K, the mutated colonies were amplified via identification primers D1-JF/D1-JR and confirmed by DNA sequencing.

In another strategy, we attempted to mutate ‘CAA’ to ‘CTA’ at position Gln139 in *nicC* gene. We developed a scarless two-step single nucleotide mutation strategy (Additional file 5). Firstly, we added 20 bp sequence ‘ATGTCTCATAAGATCATTAC’ (named A20 sequence and off-target should be avoided) between N20 sequence of pSEVA-gRic6PAM1 and its PAM sequence via inverse PCR using primers A20F/A20R. After the construction process and gene sequencing, we mutated the target single nucleotide on the homologous arm using primers Nic-SF/Nic-SR, thus creating pSEVA-NicA20. Another plasmid pSEVA-NicA21 was derived from pSEVA-NicA20 by deletion of A20 sequence in homologous arms and replaced the original N20 sequence with A20 sequence using primers NT20-F/NT20-R and NT21-F/NT21-R, respectively. After the first-step editing process, pSEVA-NicA20 was cured from the colony and another plasmid pSEVA-NicA21 was then transformed in the second step. Finally, the mutated colonies were confirmed by DNA sequencing.

### Regulation of eGFP expression intensities by dCas9

A catalytically deficient Cas9 was achieved by point mutations at two domains (D10A and H840A). For analysis of gene repression by dCas9, the relative fluorescence intensities of enhanced green fluorescent protein (eGFP) were measured by microplate reader (Thermo Varioskan™ LUX). J5 constitutive promoter [43] controlled eGFP expression, and the ribosome binding site (RBS site) was designed by RBS Calculator [44].

The dCas9 repression system consists of pCAS-ZE and pSEVA-eGFP. pCAS-ZE was derived from pCAS-RK2K-dCas9. For construction of pCAS-ZE, kanamycin marker was replaced with tetracycline marker, and the λ-Red inducible expression system was eliminated from the backbone, and the N20 sequence was replaced with new sequence, targeting J5 promoter or RBS sites of eGFP. pSEVA-eGFP was assembled by connecting eGFP expression cassette with the *Avr*I/*Eco*RI digested pSEVA644. In the eGFP expression element, a ribosome RNA binding site (5′-GCGAGCGCGATCATTCTATTAGGGAGGGAGGT-3′) was located between J5 promoter and fluorescent protein eGFP. According to CasOT, the original N20 sequence of pCAS-ZE was replaced with three new sites (by primers ZEJ-15F/ZEJ-15R, ZEJ-30F/ZEJ-30R and ZEJ-102F/ZEJ-102R respectively), and these plasmids were named as pCAS-ZE1, pCAS-ZE2, pCAS-ZE3 respectively. Another N20 sequence (TGGATCGACCTTCGTACGAG) was cloned into pCAS-ZE0 via primers ZE-J5F/ZE-J5R, which was used as the positive control. *Pseudomonas putida* KT2440 was selected as the negative control.

After transformation of these plasmids into *P. putida* KT2440 as the protocol described above, the candidate strain was screened in tetracycline and gentamycin plates and identified by the colony PCR. Next, these strain were incubated in LB medium with appropriate antibiotics, and added 10 mM rhamnose for the induction expression of gRNA cassette. Then related cells were harvested until OD_600_ reached 0.6–0.8. Using the eGFP excitation wavelength at 480 nm and fluorescence emission at 510 nm, a 200 μL of undiluted cultivated cells was added into the 96-well microtiter plates and measured the absolute fluorescence intensity (AFI) and cell density (OD_600_) by microplate reader. Finally, the fluorescence repression effect was reflected from the relative fluorescence intensity (RFI).

### Genome editing by CRISPR–Cpf1 system

To generate CRISPR–Cpf1 system, firstly, the codon optimized FnCpf1 gene (from *Francisella novicida*) was cloned from pJYS1Ptac [45] (offered by Yang Sheng) by primers pCf-1F/pCf-2R, and then Cpf1 fragment was cloned into pCAS-RK2K△Cas9 by Gibson assembly via primers pCf-2F/pCf-3R and pCf-3F/pCf-1R, resulting in pCpf1-RK2K. The 23 nt DNA sequence ‘AGGCGCAGGGCCGCTTCTTTGAG’ in pRO1600 replicon (pSEVA-gRNAT derivatives) was selected as target-curing site, which was assembled into pCpf1-RK2K (controlled by rhamnose-inducible promoter). In this FnCpf1 genome editing system, PP_3361 and PP_5301 were selected as target sites. Although FnCpf1 (5′ end ‘TTN’) and SpCas9 (3′ end ‘NGG’) recognize different PAM sequence, the repairing template can be the same donor DNA. Based on the backbone of pSEVA-gR3361T and pSEVA-gR5301T, we eliminated original Cas9-sgRNA sequence and added candidate Cpf1-crRNA into these plasmids via inverse PCR. To this end, pSEVA-gcR3361T and pSEVA-gcR5301T were constructed using primers 361-Cpf1F/361-Cpf1R and 5301-Cpf1F/5301-Cpf1R.

In this CRISPR–Cpf1 genome editing system, pCpf1-RK2K was first transformed into *P. putida* KT2440 by conjugal transfer. After KT2440 harboring pCpf1-RK2K, crRNA plasmids were transformed into this strain by electroporation. pSEVA-gR3361T or pSEVA-gR5301T was selected as a control, which was introduced into KT2440 with an equal amount of DNA (150 ng). The related electroporation conditions, genome editing process, and plasmid curing method were consistent with above CRISPR–Cas9 system.

## Results

### Establishment of a two-plasmid CRISPR–Cas9 system in *P. putida* KT2440

Various CRISPR/Cas9 systems have been established in several common microorganisms. Taking the CRISPR/Cas9 system in *E. coli* as example, it can be divided into a one-plasmid system [46], a two-plasmid system [29, 47] and a three-plasmid system [48]. Considering plasmid construction and the need for multiple genome editing rounds, we developed our system as a two-plasmid system. The *E. coli* CRISPR/Cas9 version 2.0 system developed by Jiang (unpublished) is a typical two-plasmid system that consists of several common inducible expression systems (e.g. *araBAD* expression system and *rhaBAD* expression system) and antibiotics markers used in Gram-negative bacteria. This system has also been extended into *Tatumella citrea* DSM 13699 [29]. Since *Pseudomonas* and *E. coli* are both γ-proteobacteria, many promoters and antibiotics markers share similar functions in these species. We therefore endeavored to develop a *P. putida* CRISPR/Cas9 two-plasmid system from Jiang’s *E. coli* CRISPR/Cas9 version 2.0 system.

Firstly, we replaced the replicon of pCASsac with a broad-host-range replicon (pBBR1 or RK2), creating pCAS-pBBR1 and pCAS-RK2K (Fig. 1a), respectively. To prevent sucrose to be catalyzed by SacB, we prepared electrocompetent cells with 3 mM HEPES buffer [13] instead of 300 mM sucrose [49]. In the first step, we endeavored to introduce plasmid pCAS-RK2K or pCAS-pBBR1 into KT2440. After electroporation, we could not obtain a pCAS-pBBR1 transformant. Although we identified *P. putida* KT2440 colonies harboring pCAS-RK2K, the electroporation efficiency of this plasmid was low. Toxicity of Cas9 has been reported in several bacteria [30, 45, 50], it was essential to understand whether the low transformation efficiency was caused by Cas9 toxicity, or low electroporation efficiency. Using the same electroporation parameters and an equal amount of DNA (150 ng), a series of Cas9 versions (pCAS-RK2K-nCas9D, pCAS-RK2K-nCas9H, pCAS-RK2K-dCas9, and pCAS-RK2K-Cas9FM) in pCAS-RK2K were respectively transformed into *P. putida* KT2440. As we know, different Cas9 mutation versions perform different cleavage effect or cannot cut DNA strand. In this study, different Cas9 versions made little difference to the numbers of the colony-forming units (CFU) obtained (Additional file 6). Therefore, we assumed that the low transformation efficiency obtained in the pCAS-RK2K electroporation experiments was due to low electroporation efficiency, not the lethality of Cas9. In the alternate approach, for pCAS-RK2K and its derivative plasmids, we could obtain a higher transformation efficiency by conjugation. Because the AadA proteins in Pseudomonads can degrade spectinomycin, we could use spectinomycin for the selection of *P. putida* KT2440 transconjugants [51, 52].

**Fig. 2:**
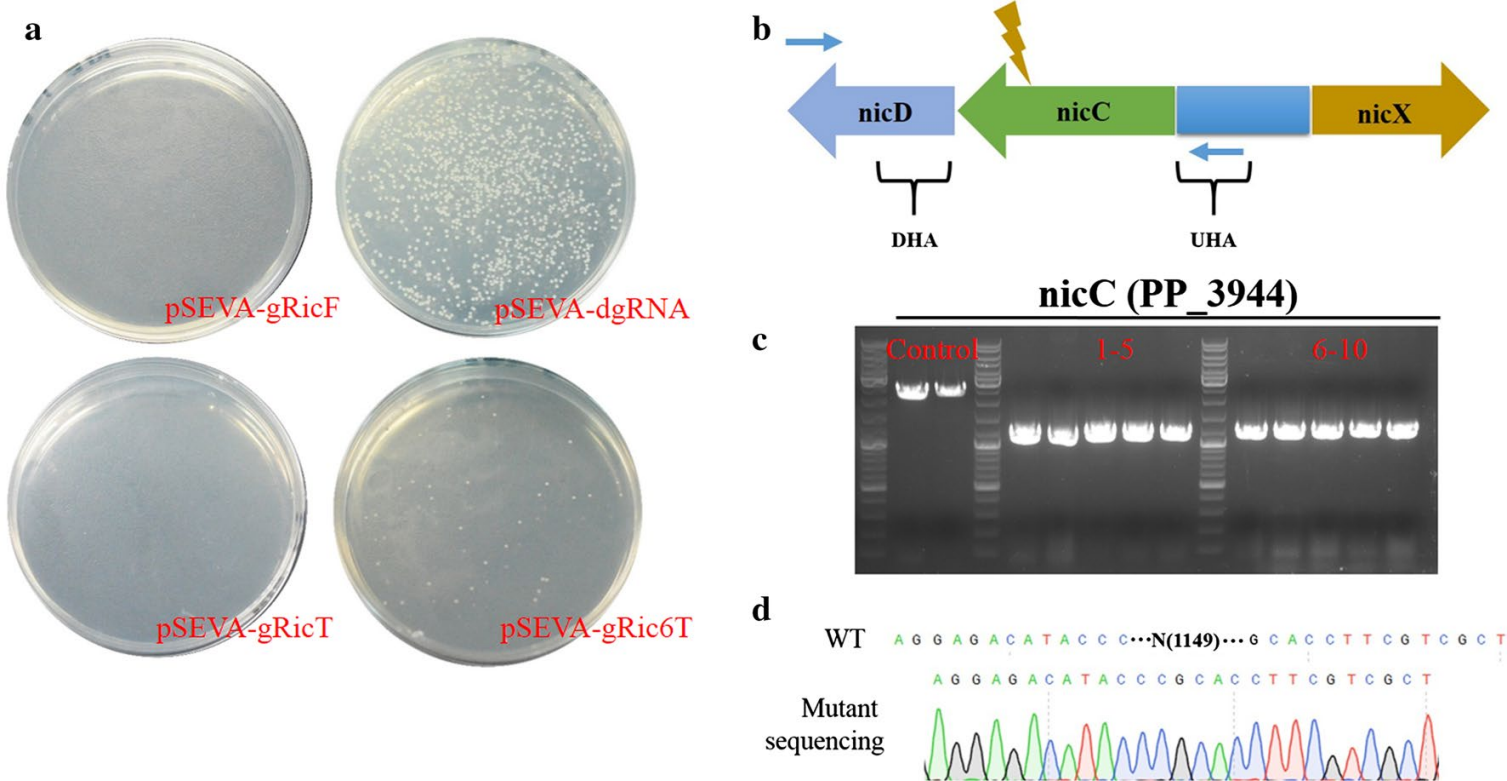
CRISPR–Cas9 mediated *nicC* gene deletion in the *Pseudomonas putida* KT2440. **a** The phenotypes of pSEVA-gRNAT derivatives transformed into KT2440 harboring pCAS-RK2K. All plasmids were electrotransformed into pCAS-RK2K cells with an equal amount of DNA. **b** The schematic represents the design of identification primers for *nicC* gene deletion. Yellow arrow means the location of N20 sequence in *nicC* gene. Blue arrow represents the location of identification primers NT-JF and NT-JR. **c** Agarose gel electrophoresis shows the result of colony PCR to confirm *nicC* gene editing efficiency. **d** DNA sequencing proves that the 1149-nt *nicC* gene have been successfully deleted

After generating *P. putida* KT2440 harboring pCAS-RK2K, we investigated whether Cas9 could be guided by sgRNA. We selected a non-essential gene (*nicC*) as the target site, and created pSEVA-gRicF and pSEVA-dgRNA. As Fig. 2a shows, almost no cells survive RNA-guided cleavage of the *Pseudomonas* genome (pSEVA-gRicF plate), but some colonies grew when Cas9 was guided by an untargeted sequence (pSEVA-dgRNA plate). However, after addition of homologous repairing arm in pSEVA-gRicT, almost no colonies could survive on the selection plates. From these results, we ascribed cell death to the off-target effects of sgRNA. Calculating the similarity between the KT2440 genome and N20 sequences of pSEVA-gRicT via CasOT, we observed that the N20 sequence was not specifically targeted to the *nicC* gene, and there were similar sequences at different locations in the genome (Additional file 7). A specific N20 sequence was designed by CasOT [42] and inserted into pSEVA-gRicT to generate pSEVA-gRic6T (N20 sequence targeted to DNA template strand). After transformation, we obtained dozens of colonies (Fig. 2a, pSEVA-gRic6T plate) and the mutation rate was 100% in 10 randomly picked strains (Fig. 2c, d). Thus, this result indicated that target gene could be mutated via our CRISPR–Cas9 system.

**Fig. 3:**
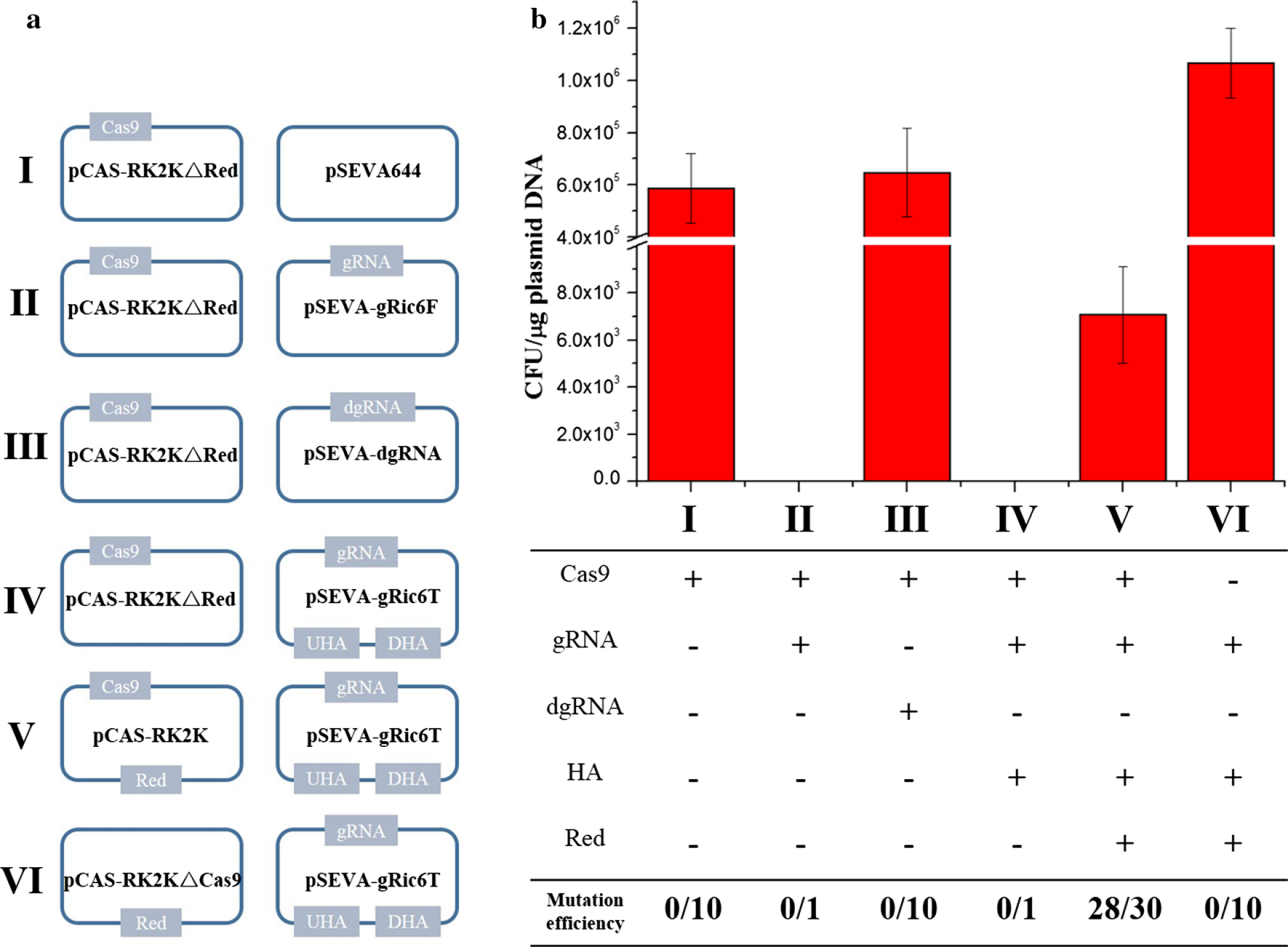
Schematic diagram of the essential effects among four components (*Cas9* protein, gRNA cassette, homologous arms and λ-Red system) in the *Pseudomonas putida* KT2440 genome editing. **a** The strategy of plasmids with different components were transformed into KT2440. pCAS-RK2K or its derivatives was first transformed into KT2440. In the second round of electroporation, pSEVA644, pSEVA-gRic6T and its derivatives were transformed into KT2440 harboring pCAS-RK2K relevant plasmids. **b** Electroporation efficiency is reflected from the total number of CFU (colony-forming units); Mutation efficiency in six groups with different components. The CFU experiment is obtained from three replicates. Cells were plated on the equal concentration antibiotics plates and the amount of DNA was equivalent in each experiment

To understand the relevance of four components—Cas9, the gRNA cassette, the homologous repairing arm, and λ-Red system—in our CRISPR/Cas9 system, we systematically constructed and transformed a series of plasmids into six groups to assess the connections among and requirements for these elements. Based on the research above, *nicC* was selected as the target site in this experiment. Based on total numbers of CFUs (Fig. 3), when Cas9 alone was introduced with pSEVA644 (Fig. 3a-I) or a non-target gRNA (Fig. 3a-III), we obtained a large number of transformants. Testing an otherwise intact CRISPR/Cas9 system without Cas9 protein (Fig. 3a-VI), the number of colonies was slightly higher than for the two previous groups, indicating that the presence of Cas9 may exert slight cell toxicity or decrease the electroporation efficiency of small plasmids. Next, without the heterologous repairing ability provided by the λ-Red system (Fig. 3a-II, IV), we found cells hardly survived the Cas9-induced DSBs using their endogenous repairing system, even on addition of a homologous repairing template (Fig. 3a-IV). In conclusion, only when the CRISPR/Cas9 system contains its four essential components (Fig. 3a-V) could efficient genome editing be achieved.

### Analysis of multigene editing efficiencies

To further investigate the utility of CRISPR/Cas9 system in *P. putida* KT2440, we performed different genomic modification approaches in *nicC* gene site (Table 1). Firstly, it is essential to know the effect of N20 sequence targeting different DNA template strand. We designed pSEVA-gRic5T, which was a plasmid with a new N20 sequence targeting the DNA non-template strand. After electroporation of pSEVA-gRic5T, we picked 15 colonies from plates and found that the mutation rate reached 93.3%. Thus, for the same target gene, a similar high mutation rate was observed when either DNA strand was targeted. Meanwhile, we also examined gene replacement of DNA fragment of three different lengths of (Table 1). All these fragments were respectively inserted between the upstream and downstream homologous arm of pSEVA-gRic6T, and transformed into *P. putida* KT2440 harboring pCAS-RK2K using genome editing protocols described above. These insertions all showed a high knock-in rate (Table 1), which indicated that the length of the inserted fragment (within the range we tested) did not decrease the mutation rate.

**Table 1:** Mutation efficiency of the type II CRISPR system in *P. putida* KT2440

Host cell	gRNA plasmid	Deletion	Replacement	Insertion	Size	Results	Plasmid curing efficiency
KT2440 harboring pCAS-RK2K	pSEVA-gRic6T	*nicC* (PP _3944)			1149	15/15	ND
KT2440 harboring pCAS-RK2K	pSEVA-gRic5T	*nicC* (PP _3944)			1149	39/45	ND
KT2440 harboring pCAS-RK2K	pSEVA-gRic6TΔNicC::rhla	*nicC* (PP _3944)	*rhla*		888	38/40	ND
KT2440 harboring pCAS-RK2K	pSEVA-gRic6TΔNicC::dCas9	*nicC* (PP _3944)	dCas9		4107	20/20	ND
KT2440 harboring pCAS-RK2K	pSEVA-gRic6TΔNicC::T7 RNA polymerase	*nicC* (PP _3944)	T7		4518	14/20	5/5
KT2440 harboring pCAS-RK2K	pSEVA-gRic6PAM2	Single nucleotide	Single nucleotide		1	5/5	ND
KT2440 harboring pCAS-RK2K	pSEVA-NicA20	Single nucleotide	Single nucleotide	A20	21	7/7	ND
KT2440 harboring pCAS-RK2K	pSEVA-NicA21	A20			20	5/5	ND
KT2440 harboring pCAS-RK2K	pSEVA-gR0552T	PP_0552			1089	8/10	ND
KT2440 harboring pCAS-RK2K	pSEVA-gR3361T	PP_3361			3048	11/13	ND
KT2440 harboring pCAS-RK2K	pSEVA-gR3733T	PP_3733			1059	18/18	ND
KT2440 harboring pCAS-RK2K	pSEVA-gR3889T	PP_3889			1299	11/12	ND
KT2440 harboring pCAS-RK2T	pSEVA-gR3947-3948T	PP_3947–PP_3948			4033	18/18	5/5
KT2440 harboring pCAS-RK2T	pSEVA-gR3939-3940T	PP_3939–PP_3940			2743	9/10	5/5
KT2440 harboring pCAS-RK2T	pSEVA-gR3846T	PP_3846			816	10/10	ND
KT2440 harboring pCAS-RK2T	pSEVA-gR1706T	PP_1706			363	15/16	ND
KT2440 harboring pCAS-RK2T	pSEVA-gR5301T	PP_5301			264	10/10	ND
KT2440 harboring pCpf1-RK2K	pSEVA-gcR3361T	PP_3361			3048	4/4	ND
KT2440 harboring pCpf1-RK2K	pSEVA-gcR5301T	PP_5301			264	9/9	2/2

*rhla* was cloned from *Pseudomonas aeruginosa* PAOl, and T7 RNA polymerase was amplified from *Escherichia coli* BL21(DE3)

*ND* not determined

**Fig. 4:**
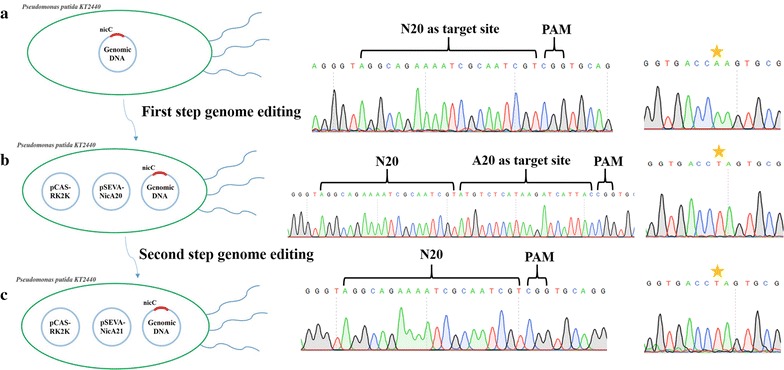
The two-step strategy of single nucleotide mutation for PAM unavailability sites using CRISPR–Cas9 system. **a** The N20 sequence from pSEVA-NicA20 was used as Cas9 cutting site in KT2440, and the Gln139 in *nicC* gene was targeted as mutation site by mutating CAA to CTA. Yellow star means the target nucleotide. **b** After the first step genome editing, an added artificial N20 sequence (A20 sequence) and single nucleotide mutation (At Gln139 by mutating CAA to CTA) in pSEVA-NicA20 homologous arm were inserted into KT2440 genome. **c** Through the first step, single nucleotide mutation was applied into target locus. Then, we attempted to eliminate A20 sequence by curing pSEVA-NicA20 and using pSEVA-NicA21 for next genome editing step. In pSEVA-NicA21, the target site was changed from N20 sequence to A20 sequence. In the homologous arm, A20 sequence was eliminated and single nucleotide mutation was retained

In addition, we achieved single nucleotide mutation (SNM) in *nicC* gene, by changing the PAM sequence ‘CGG’ to ‘CAG’ via pSEVA-gRic6PAM2 (Table 1). Nevertheless, except for the PAM position, the editing of PAM unavailability sites was not so simple [53]. In order to achieve single nucleotide mutation for PAM unavailability sites, we need to select an ideal N20 sequence which can satisfy both non-off-target in genome and silent mutation (avoid repairing template degradation) will not change amino acid sequence. Next, we attempted to modify the Gln139 in *nicC* gene to Leu139 by mutating the codon ‘CAA’ to ‘CTA’. In this experiment, we established a scarless two-step replacement strategy for SNM (Additional file 5), thus making a silent mutation in repairing template was not required. An A20 sequence was designed to insert between the N20 sequence and the PAM motif in pSEVA-gRic6PAM1, and this operation could result in replacement of the PAM motif of the original N20 sequence with the A20 sequence. In the first-step editing process, the target nucleotide was mutated and the A20 sequence was inserted into the genome (Fig. 4b). Next, after curing of the pSEVA-NicA20, the A20 sequence was eliminated from the genome by transformation with pSEVA-NicA21. Finally, the single nucleotide mutation was retained in the target site (Fig. 4c).

Aside from these versatile mutations of *nicC* gene, nine other target sites, PP_0552, PP_3361, PP_3733, PP_3889, PP_3939–PP_3940, PP_3947–PP_3948, PP_1706, PP_3846 and PP_5301, were used to further examine the mutation rate of the CRISPR/Cas9 system. PP_0552, PP_3361, PP_3733 and PP_3889 were selected as genome editing sites in KT2440 harboring pCAS-RK2K. After transformation of pSEVA-gRNAT derivative plasmids (pSEVA-gR0552T, pSEVA-gR3361T, pSEVA-gR3733T and pSEVA-gR3889T) into KT2440 harboring pCAS-RK2K, we obtained high mutation rates in every case. The resulting mutation efficiencies for PP_0552, PP_3361, PP_3733 and PP_3889 were 80% (8/10), 84.6% (11/13), 100% (18/18) and 91.6% (11/12), respectively. Having demonstrated the mutation efficiency of KT2440 harboring pCAS-RK2K, the utility of pCAS-RK2T (tetracycline version) was examined in another five sites (PP_3947-PP_3948, PP_3939-PP_3940, PP_3846, PP_1706 and PP_5301). After electroporation of pSEVA-gR3947-3948T into KT2440 harboring pCAS-RK2T, all eighteen mutant clones were proved to be successful editing. In the case of PP3939-PP3940, nine out of ten transformants were mutated. When pSEVA-gR3846T was introduced into KT2440 containing pCAS-RK2T, the editing efficiency of PP_3846 was 100% (10/10), similar to the frequency (18/18) obtained from PP_3947–PP_3948 mutation experiment. For PP_1706 and PP_5301, the colony PCR indicated that mutation efficiencies of 93.7% (15/16) and 100% (10/10) were generated in relevant experiment. Comparison of the deletion rate at the nine different locations is given in Table 1. The mutation rate varied between sites, but the editing efficiency overall was high.

### Interference the expression of eGFP by dCas9

To expand the function of our CRISPR/Cas9 system, we endeavored to establish a CRISPR interference (CRISPRi) system in *P. putida* KT2440. In CRISPRi, a nuclease deficient Cas9 (dCas9) can be guided by a sgRNA or multi sgRNAs to exert transcriptional repression upstream of target genes. Without its endonucleolytic activity, dCas9 remains bound to the target locus; this has been validated for regulation of gene expression in several organisms [27, 28, 50].

**Fig. 5:**
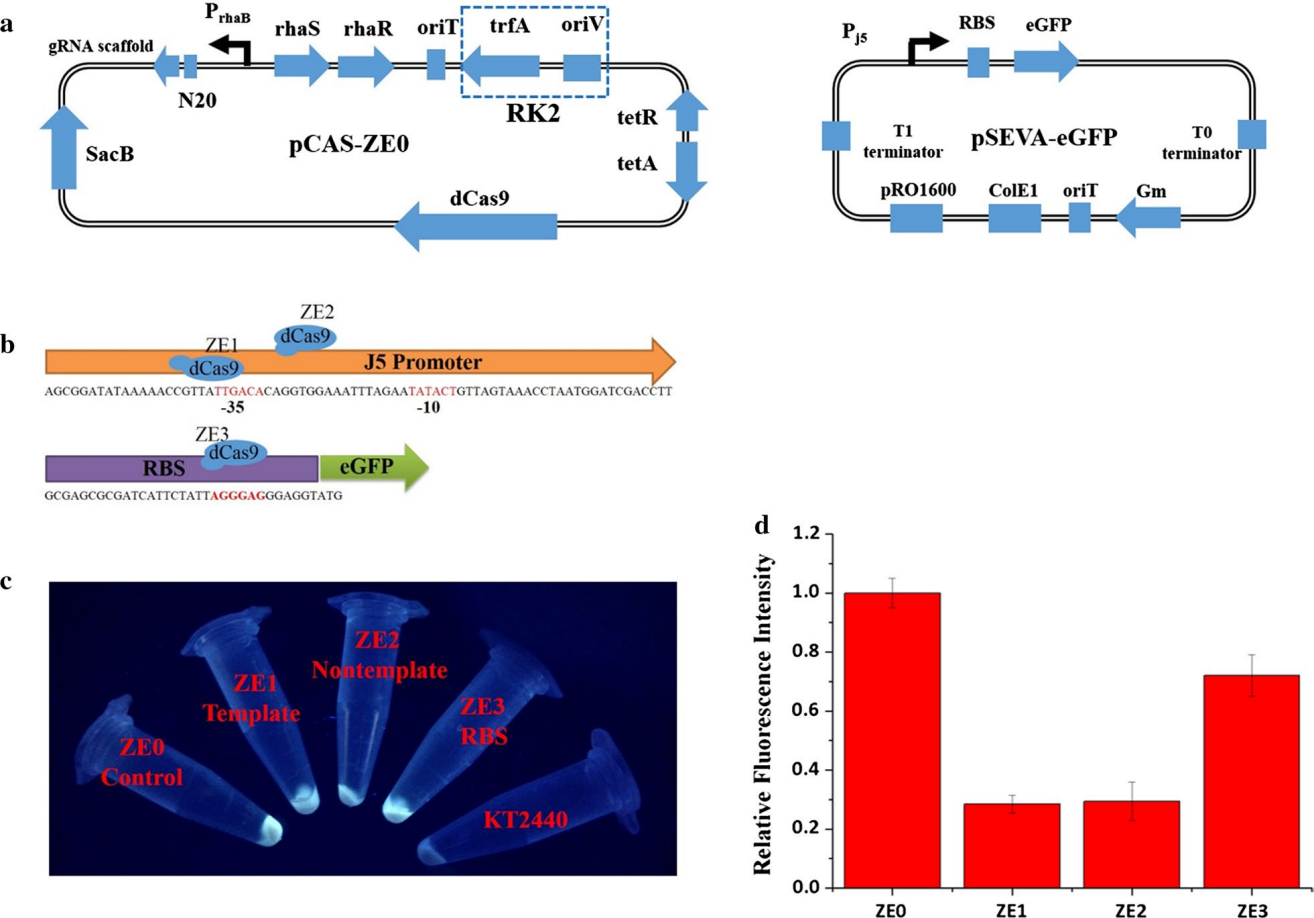
CRISPR–dCas9 mediated transcription inhibition in the *Pseudomonas putida* KT2440. **a** Schematic representation of pCAS-ZE0 and its derivatives (pCAS-ZE1, pCAS-ZE2, pCAS-ZE3) used for transcription inhibition. Plasmid-borne enhanced green fluorescence protein (eGFP) was selected as target site. **b** Illustration of different dCas9 binding sites are indicated in the upstream sequence of plasmid pSEVA-eGFP. ZE1 and ZE2 were targeting the − 35 region of J5 promoter, and ZE3 was binding with Ribosome Binding Site (RBS). To examine the effect of selecting different DNA strand, ZE1 was designed to bind with template strand and ZE2 was located at the non-template strand. **c** KT2440 cells harboring dCas9 and eGFP plasmids were gathered with equal amount and exposed under UV light. Blank KT2440 cells were used as control. **d** Comparsion of the repression effectiveness of dCas9 binding with different target sites

We attempted to demonstrate a CRISRPi effect via the expression intensity of enhanced green fluorescent protein (eGFP) in pSEVA-J5-eGFP (Fig. 5a). Three sgRNAs targeted to different loci of eGFP transcription were designed and inserted into pCAS-ZE1, pCAS-ZE2, and pCAS-ZE3 respectively (Fig. 5b). After two-step electrotransformation or co-electrotransformation, KT2440 harboring dCas9 and eGFP was screened and incubated in LB medium containing antibiotics and rhamnose. After overnight cultivation, the cell concentration (OD_600 nm_) and the absolute fluorescence intensity (AFI) were measured simultaneously in a microplate reader. The final fluorescence repression results were evaluated in terms of relative fluorescence intensity (RFI) (Fig. 5d), which was calculated as AFI divided by OD_600_. Compared to the pCAS-ZE0 control group (N20 sequence lacked target site), approximately 350% and 340% fluorescence repression effects were obtained in groups ZE1 (N20 sequence targeted to the template strand) and ZE2 (N20 sequence targeted to the nontemplate strand) when the sgRNA associated with dCas9 binding to the − 35 position in the J5 promoter, on either DNA strand. Only a slight repression effect (138%) was achieved in the group ZE3 (N20 sequence targeted to RBS sites on the nontemplate strand). As for groups ZE1 and ZE2, similar repression effects were achieved by targeting different DNA template strands when dCas9 targeted to − 35 region in the promoter. This repression efficiency indicated that dCas9 exhibited no strand bias to − 35 region in the promoter. In case of group ZE3, the repression activity of dCas9 was significantly decreased compared to groups ZE1 and ZE2. These results demonstrated that a different dCas9-binding site (promoters or RBS sites) led to distinct repression of intensity. Because of the obvious fluorescence of eGFP, we could also observe transcriptional blocking by the naked eyes. Samples from each group were concentrated in respective 1.5-mL Eppendorf tubes and exposed to UV light. The results (Fig. 5c) were fully consistent with the RFI parameters. Altogether, these data demonstrate that dCas9 could be an efficient tool for gene repression in *P. putida* KT2440.

### Application of CRISPR–Cpf1 genome editing system in *P. putida* KT2440

We also endeavored to explore the feasibility of using FnCpf1 for genome editing in *P. putida* KT2440. We found that pCpf1-RK2K could only be transferred into KT2440 by conjugal transfer. Although we obtained dozens of clones by electroporation, pCpf1-RK2K could not be identified in these cells.

**Fig. 6:**
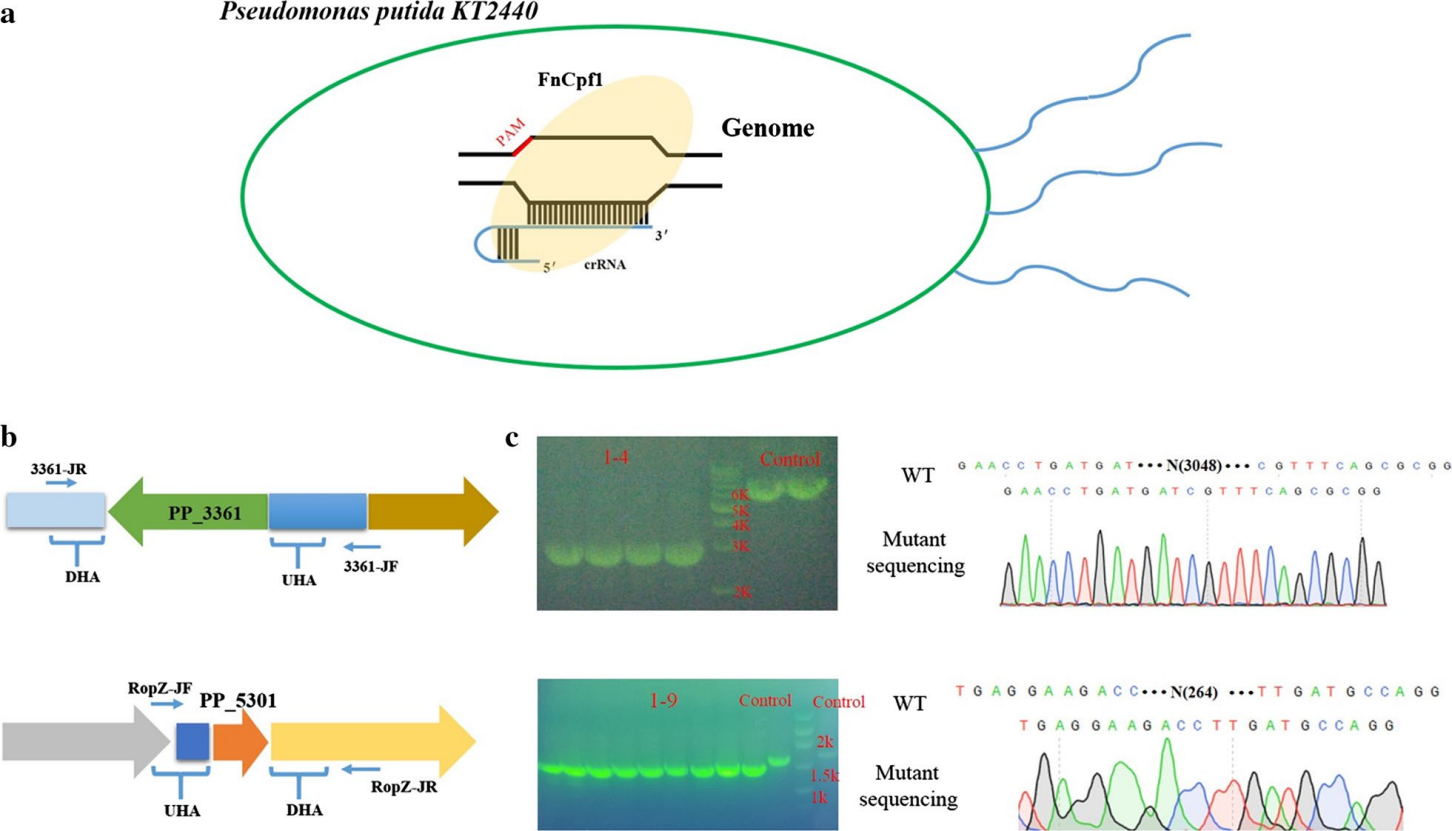
CRISPR–Cpf1 mediated genome editing in the *Pseudomonas putida* KT2440. **a** Overview of the genome editing by CRISPR–Cpf1 in *P. putida* KT2440. **b** The schematic showing the design of identification primers to confirm genome editing in KT2440. **c** Agarose gel electrophoresis and the result of DNA sequencing show that pCpf1-RK2K enables gene deletion in KT2440

After preparation of electrocompetent KT2440 harboring pCpf1-RK2K, plasmids pSEVA-gcR3361T, pSEVA-gcR5301T and control plasmids (pSEVA-gR3361T and pSEVA-gR5301T) were transformed by electroporation, respectively. Using the same procedure as for Cas9 genome editing, several random cells were picked from plates and tested by colony PCR. Using this CRISPR–Cpf1 system, we achieved deletion rate of 100% in PP_3361 (Fig. 6b), and PP_5301 (Fig. 6b). Thus, we preliminarily demonstrated that FnCpf1 can be harnessed as a genome editing tool in *P. putida* KT2440.

## Discussion

**Fig. 7:**
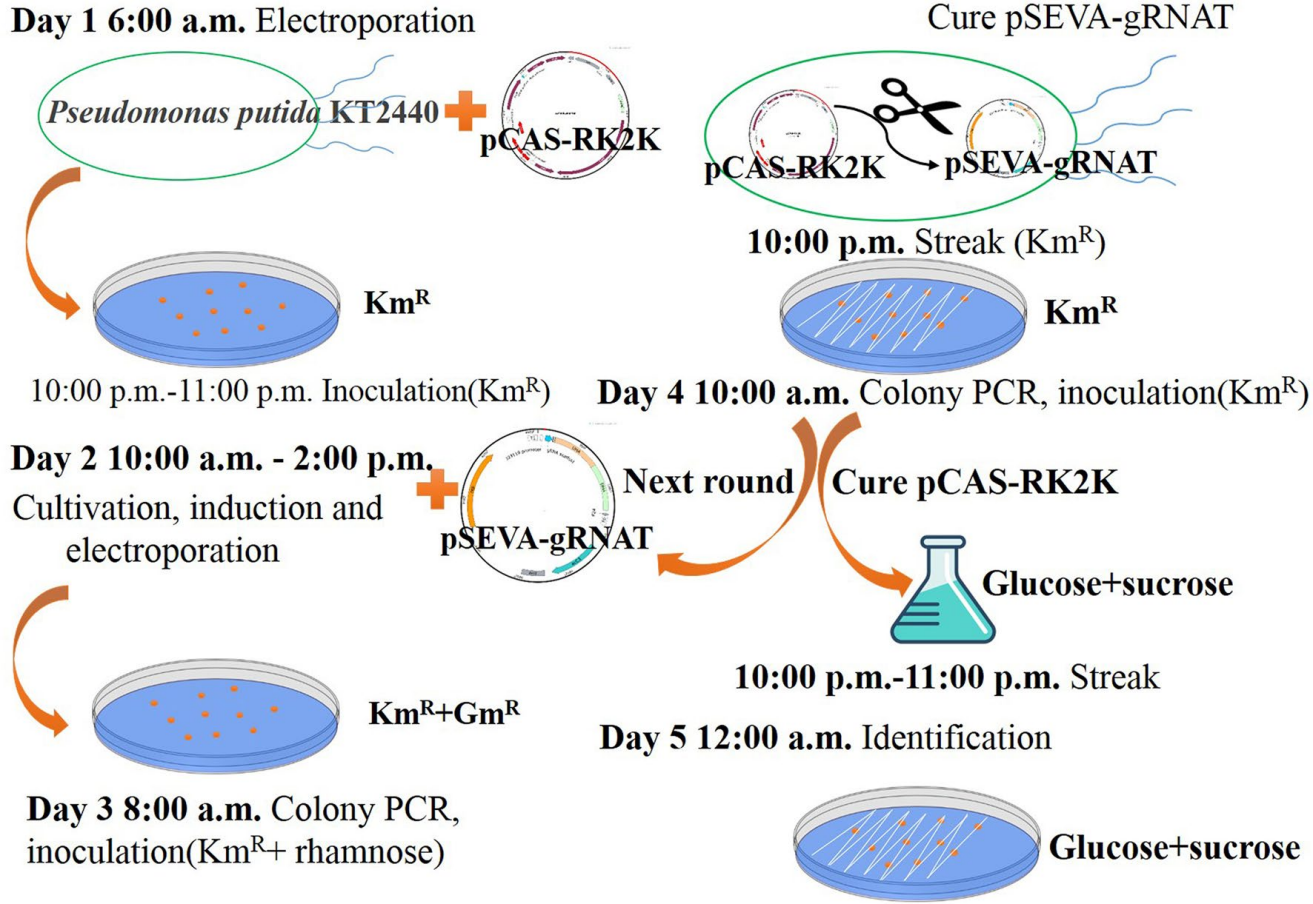
Diagram for the CRIPSR–Cas9-assisted genome editing in *P. putida* KT2440. Day 1: Introduce the pCAS-RK2K plasmid into *P. putida* KT2440, and then inoculate the transformants in LB medium overnight; Day 2: Transfer the cultivated cells into fresh LB medium and add arabinose to trigger expression of λ-Red proteins. Next, cells were prepared as elecompetent cells and the pSEVA-gRNAT plasmids were transferred to KT2440 harboring pCAS-RK2K. Day 3: Screen out the mutants by colony PCR. The mutants were inoculated in LB medium containing antibiotic and rhamnose in the morning; Streak the cultivated cells on LB agar in the evening; Day 4: Screen out the mutants that have lost the pSEVA-gRNAT plasmids, and inoculate the mutants in LB medium containing glucose and sucrose. Next, in the evening, the cultivated cells were streak on plate containing glucose and sucrose, and then cultivated overnight. The mutants that have been cured of pSEVA-gRNAT can be used for the next round of genome editing; Day 5: Identify the mutants from the selection plate

In this study, we have demonstrated that a CRISPR/Cas9 system can be used for genome editing and regulation of gene expression in *P. putida* KT2440. A two-plasmid CRISPR/Cas9 system [29] was selected as our backbone because of the need for continual genome editing. In contrast to previous genome editing approaches [5–17] and a recently reported CRISPR–Cas9 method [37] (Table 2), our CRISPR/Cas9 system prove to be an efficient and fast tool in *P. putida* KT2440. The CRISPR/Cas9 genome editing system in *P. putida* is first developed by Aparicio et al. [37], in which gene deletion is performed by Cas9 protein assisting with single strand DNA (ssDNA) and a recombinase protein (Ssr) in a three-plasmid system. However, the genome editing efficiency in this approach has highly variable efficiency (13–93.2% below 5 kb target), gene insertion and replacement cannot be realized, and a plasmid curing strategy is also suboptimal. In our CRISPR/Cas9 system, genome editing efficiency reached more than 70%, and the total process including gene editing and plasmid curing could be achieved within 5 days (Fig. 7), which outcompetes most previous approaches in *Pseudomonas*. Owing to the high efficient plasmid-curing strategy, for continual genome editing, we can perform three rounds of genome editing in 1 week (Additional file 8). Besides gene deletion and insertion, transcriptional repression caused by dCas9 via different expression intensities of eGFP was successfully demonstrated in our study. In addition, we established another type II CRISPR system in KT2440, the CRISPR–Cpf1 system, which showed high genome editing efficiency.

**Table 2:** Comparison of different genetic editing tools in *Pseudomonas*

Number	Method	Time spent	Scarless	Markerless	Insertion	Exact locus	Mutation efficiency	Convenient continual genome editing	Efficient plasmid curing	References
1	This work	5 days	Yes	Yes	Yes	Yes	70–100%	Yes	Yes	
2	CRISPR–Cas9 Ssr	4–5 days	Yes	Yes	ND	Yes	13–93%	No	No	[37]
3	I-SceI	5–6 days	Yes	Yes	ND	Yes	14–84%	No	No	[14]
4	Tn5 FLP-FRI	2 weeks	No	No	No	No	ND	No	No	[17]
*5*	Tn5/Tn7	l week	No	No	Yes	No	ND	No	No	[15, 16]
6	Flp/FRT SacB	5–6 days	No	Yes	ND	Yes	ND	No	No	[9]
7	λ-Red SacB	4–5 days	Yes	Yes	ND	Yes	88–98%	No	No	[12]
8	Cre-lox λ-Red	4–6 days	No	Yes	ND	Yes	70–100%	No	No	[13]
9	pyrF	2 weeks	No	No	ND	Yes	ND	No	No	[6]
10	upp	5–6 days	Yes	Yes	Yes	Yes	10–40%	No	No	[7]
11	Red/ET	4–5 days	No	No	Yes	Yes	ND	No	No	[11]

*ND* not determined

To establish a new genome editing approach in *P. putida* KT2440 via the CRISPR–Cas9 system, we first considered the transformation method for our plasmids. In our study, we applied two broad-host-range replicons for pCASsac function in *Pseudomonas*: pCAS-pBBR1 could not be transformed into hosts via electroporation, and the electroporation efficiency of pCAS-RK2K was low. Due to previous reports of Cas9 toxicity in several bacteria [30, 45, 50], we constructed five Cas9 protein versions that were transformed into *P. putida* KT2440 for toxicity analysis. There was no obvious difference of transformation efficiency between the five Cas9 versions, thus we could eliminate Cas9 toxicity as a major issue.

In the next sgRNA construction step, we demonstrated Cas9 protein could be guided by sgRNA, which killed almost all the cells in the *nicC* plates. In the following procedures, however, we could hardly obtain any transformants from screening plates even with addition of donor DNA and the help of heterologous recombination proteins. Obviously, off-target effect of Cas9 was another challenge in this study, and could cause Cas9 to target other locations in the genome. Without a homologous repairing template, *Pseudomonas* cells could hardly survive multiple genome breaks. By means of CasOT, we could design sequence to avoid cells being cut by Cas9 at off-target sites. Finally (Fig. 2), transformants screened from plates showed a high knock-out efficiency. Due to the relative lower frequencies of native homology-directed repair system in *P. putida KT2440*, λ-Red-mediated recombination was essential for genome editing to proceed (Fig. 3). Additionally, we tried to simplify the construction of pSEVA-gRNAT plasmids, e.g. the homologous repairing arm supplied as a fragment was co-transformed with pSEVA-gRNAF (lacked the homologous repairing arm) into KT2440 harboring pCAS-RK2K. Although we could obtain dozens of transformants on plates, the mutation rate of target site dramatically decreased to 10%. In previous other genome editing methods [14], we found donor DNA was inserted into plasmids, and we assumed this phenomenon might be correlated with a lower efficiency of DNA uptake in *P. putida* KT2440.

After the preliminary establishment of the CRISPR/Cas9 system, we attempted different genome editing approaches in *P. putida* KT2440. We showed that an N20 sequence could obtain a similar high mutation rate irrespective of the target DNA template strand, and the efficiency of fragment insertion was not affected by the length of the fragment (within the range we tested). In addition, we developed a single nucleotide mutation approach for different target site conditions. A One-step method could be performed if the desired site is in a PAM motif or the last 12 bp of the N20 sequence near a PAM region [53]. However, not every nucleotide satisfies these requirements, and for those we developed a two-step strategy. We designed a high-specific 20-bp sequence (A20 sequence) between the N20 sequence and PAM motif, which could avoid the degradation of donor DNA. After the first step, single nucleotide could be mutated and the A20 sequence left in the genome. Then, in the next step, we changed N20 sequence to this A20 site, and then A20 sequence was eliminated from homologous repairing template. By this method, we could obtain a scarless single nucleotide modified strain.

Cas9 can be guided by multi sgRNAs, and multi-gene editing performed by CRISPR/Cas9 is a unique feature compared with other genome editing tools. In our study, we also attempted to perform a double-locus editing experiment, but could not obtain a strain with double-locus deletion after we picked more than 100 transformants in one experiment. Although gene deletion simultaneously performed in two sites was successfully demonstrated in a recent study [37], the editing efficiency of this method was extremely low (0.5%). In our study, we do not recommend this method because of its low efficiency and time-consuming plasmid construction. Instead, we tended to perform multigene editing by multiple rounds, achieving genomic mutation in a single target site in each round. This approach was highly efficient, and the manipulation time was reduced via the flexible and efficient curing strategy in our two-plasmid system.

CRISPR interference is an alternative approach to gene knock-out for regulation of gene expression and has been widely used in bacteria such as *E. coli* [54] and *Clostridium* [50]. Here, we selected eGFP and tested different dCas9 binding sites as target sites for CRISPRi. Different dCas9 target sites had a significant impact on the level of transcriptional repression, but the DNA strand on which an N20 sequence was located had no obvious effect on dCas9 blocking of gene expression. When dCas9 targeted to the starting region of promoter, this repression effect was much better than RBS sites. The exploration of pCAS-ZE systems could be a useful approach for metabolic engineering regulation and other genomic manipulation in *P. putida* KT2440.

The CRISPR–Cpf1 system is an emerging Type II CRISPR genome editing tool, which has also been established in several organisms [45, 55, 56]. Due to its longer target sequence (23–25 nt), Cpf1 exhibits relatively low potential for off-target activity, creating applicability of CRISPR–Cpf1 as a complementary genome editing tool to CRISPR–Cas9 system [38]. The establishment in the study of the CRISPR–Cpf1 system in strain KT2440 will be an alternative tool for some gene loci, for which it is hard to obtain a non-off-target N20 sequence.

## Conclusions

Here, high efficient and versatile functions CRISPR/Cas9 system have been successfully established in *P. putida* KT2440. Although off-target effects of Cas9 are a challenge in target locus selection, versatile gRNA selection tools [42, 57, 58] and an enhanced-specificity mutant version of Cas9 have been developed [59]; these tools can minimize off-target effects and increase on-target possibilities. Besides *P. putida*, there are abundant *Pseudomonas* species, such as *P. aeruginosa*, a commonly used strain in immunology [60], and *P. fluorescens*, which possess growth-promoting ability for host plant [61]. Currently, we are trying to extend the CRISPR/Cas9 system and the CRISPR/Cpf1 system into *P. aeruginosa* PA01 and *P. fluorescens* Pf0-1 in our laboratory. *P. putida* KT2440 is a well-known “generally recognized as safe” (GRAS) bacterium, a versatile platform in biotechnology [62], and an important next generation synthesis biology chassis [63]. Although the complete genome of *P. putida* KT2440 was sequenced [64] and revisited [65], and a set of bioinformatics tools [66, 67] was also developed, the practical biotechnological methods for predictive verification and modification of these chassis cells are suboptimal. Thus, the establishment of CRISPR–Cas9 and CRISPR–Cpf1 systems in strain KT2440 will spur and facilitate further understanding and application of this strain, and form the basis to extend CRISPR genome systems into other important *Pseudomonas* strains.

## Additional files

**Additional file 1.** The strains and plasmids used in this research.

**Additional file 2.** Key primers used in this study.

**Additional file 3.** The construction strategy of pCAS-RK2K and pCAS-pBBR1.

**Additional file 4.** PAM-guide sequences used in related plasmids.

**Additional file 5.** Plasmids of two-step single nucleotide mutation strategy.

**Additional file 6.** The total CFU calculation from different Cas9 versions.

**Additional file 7.** The analysis of sgRNA off-target in KT2440 by CasOT.

**Additional file 8.** Flow-chart of 3 rounds of continual genome editing in *P. putida* KT2440.

## Abbreviations

CFU: colony-forming units
CRISPRi: clustered regularly interspaced short palindromic repeat interference
crRNA: CRISPR RNA
DSB: double-stranded breaks
DHA: downstream homologous arm
dCas9: nuclease-deficient Cas9
eGFP: enhanced green fluorescent protein
GRAS: generally regarded as safe
LB: Luria–Bertani
PAM: protospacer-adjacent motif
RBS: ribosome binding site
SNM: single nucleotide mutation
sgRNA: single-guide RNA
UHA: upstream homologous arm
