# Supplementary material for: Genome editing and transcriptional repression in Pseudomonas putida KT2440 via the type II CRISPR system
Source: Microb Cell Fact. 2018 Mar 13;17:41. doi: 10.1186/s12934-018-0887-x (PMC5851096; doi:10.1186/s12934-018-0887-x)
Supplement: Supplementary file 1 — Additional file 1. The strains and plasmids used in this research. [file 12934_2018_887_MOESM1_ESM.docx]

**Additional file 1. The strains and plasmids used in this research**

| **TABLE 1 Bacteria and plasmids used in this study** | | |
| --- | --- | --- |
| **Strains or plasmid** | **Relevant characteristics** | **Source** |
| **Strains** |  |  |
| E. coli DH5α | F- Φ80lacZΔM15, hsdR17 (rk-mk+)λ-, Δ(lacZYA-argF), U169, recA1, endA1, thi-1, gyrA96, relA, deoR, nupG,glnV44 | Lab stock |
| E. coli S17-1 | res−, proA, mod+, thi-1, integrated copy of RP4, | Lab stock |
| Pseudomonas putida KT2440 | mt-2 derivative clearance of the TOL plasmid pWW0 | Lab stock |
| **Original Plasmids** |  |  |
| pCASsac | oripSC101, Km^R^, Pcas-Cas9, ParaB-Red, PrhaB-sgRNA-pMB1, SacB | Offered by Yang Sheng |
| pTargetF | oripMB1, Spe^R^, Pj23119-sgRNA-pMB1 | Offered by Yang Sheng |
| pBBR1MCS2 | oripBBR1, Km^R^, lacZ | Lab stock |
| pSEVA258 | oriRSF1010, Km^R^, xylS-Pm | SEVA Datebase |
| pSEVA429 | oriRK2, Sm^R^, alkS-PalkB | SEVA Datebase |
| pSEVA644 | oripRO1600/ColE1, Gm^R^, lacIq-Ptrc | SEVA Datebase |
| pET30a-eGFP | oripBR322, orif1, Km^R^, PT7-eGFP | Lab stock |
| pJYS1Ptac | ori pSC101, Km^R^, Ptac-recT, PlacM-Cpf1 | Offered by Yang Sheng |
| **CRISPR/Cas9 plasmids** |  |  |
| pCAS-pBBR1 | oripBBR1, Km^R^, Pcas-Cas9, ParaB-Red, PrhaB-sgRNA-pRO1600, SacB | This work |
| pCAS-RK2K | oriRK2, Km^R^, Pcas-Cas9, ParaB-Red, PrhaB-sgRNA-pRO1600, SacB | This work |
| pCAS-RK2T | oriRK2, Tet^R^, Pcas-Cas9, ParaB-Red, PrhaB-sgRNA-pRO1600, SacB | This work |
| pCAS-RK2-nCas9D | oriRK2, Km^R^, Pcas-nCas9D, ParaB-Red, PrhaB-sgRNA-pRO1600, SacB | This work |
| pCAS-RK2-nCasH | oriRK2, Km^R^, Pcas-nCas9H, ParaB-Red, PrhaB-sgRNA-pRO1600, SacB | This work |
| pCAS-RK2-dCas9 | oriRK2, Km^R^, Pcas-dCas9, ParaB-Red, PrhaB-sgRNA-pRO1600, SacB | This work |
| pCAS-RK2-Cas9FM | oriRK2, Km^R^, Pcas-Cas9FM, ParaB-Red, PrhaB-sgRNA-pRO1600, SacB | This work |
| pCAS-RK2△Cas9 | oriRK2, Km^R^, ParaB-Red, PrhaB-sgRNA-pRO1600, SacB | This work |
| pCAS-RK2△Red | oriRK2, Km^R^, Pcas-Cas9, PrhaB-sgRNA-pRO1600, SacB | This work |
| pSEVA258-Cas9FM | oriRSF1010, Km^R^, Pcas-Cas9FM, ParaB-Red, PrhaB-sgRNA-pRO1600, SacB | This work |
| pSEVA-gRNA | oriPRO1600/ColE1, Gm^R^, Pj23119-sgRNA-pMB1 | This work |
| pSEVA-gRicT | Derived from pSEVA-gRNA, Pj23119-sgRNA-nicC(PP_3944), homologous arms | This work |
| pSEVA-gRicF | Derived from pSEVA-gRNA, Pj23119-sgRNA-nicC(PP_3944) | This work |
| pSEVA-dgRNA | Derived from pSEVA-gRNA, Pj23119-sgRNA-dgRNA | This work |
| pSEVA-gRic5T | Derived from pSEVA-gRNA, Pj23119-sgRNA-nicC(PP_3944), homologous arms(two 0.5kb arms) | This work |
| pSEVA-gRic6F | Derived from pSEVA-gRNA, Pj23119-sgRNA-nicC(PP_3944) | This work |
| pSEVA-gRic6T | Derived from pSEVA-gRNA, Pj23119-sgRNA-nicC(PP_3944), homologous arms(two 0.5kb arms) | This work |
| pSEVA-gRic6T△NicC::RhaA | Derived from pSEVA-gRic6T, homologous arms(RhaA was added between upstream and downstream arms) | This work |
| pSEVA-gRic6T△NicC::dCas9 | Derived from pSEVA-gRic6T, homologous arms(dCas9 was added between upstream and downstream arms) | This work |
| pSEVA-gRic6T△NicC::T7 | Derived from pSEVA-gRic6T, homologous arms(T7 was added between upstream and downstream arms) | This work |
| pSEVA-gRic6PAM1 | Derived from pSEVA-gRic6T, homologous arms(500bp upstream of nicC linked with the first 500bp of nicC) | This work |
| pSEVA-gRic6PAM2 | Derived from pSEVA-gRic6T, homologous arms(500bp upstream of nicC linked with the first 500bp of nicC, a single nucleotide mutated in PAM motif) | This work |
| pSEVA-NicA20 | Derived from pSEVA-gRic6PAM1, homologous arms(A20 sequence was added between N20 sequence and PAM motif, a single nucleotide mutated in) | This work |
| pSEVA-NicA21 | Derived from pSEVA-NicA20, Pj23119-sgRNA-A20, homologous arms(A20 sequence was eliminated between N20 sequence and PAM motif, a single nucleotide mutated in) | This work |
| pSEVA-2gRNA-3733-3361 | Derived from pSEVA-gRNA, Pj23119-sgRNA-PP_3361, Pj23119-sgRNA-PP_3733, homologous arms(four 1.0 kb arms) | This work |
| pSEVA-gR0552T | Derived from pSEVA-gRNA, Pj23119-sgRNA-PP_0552, homologous arms(two 0.5 kb arms) | This work |
| pSEVA-gR3361T | Derived from pSEVA-gRNA, Pj23119-sgRNA-PP_3361, homologous arms(two 1.0 kb arms) | This work |
| pSEVA-gR3733T | Derived from pSEVA-gRNA, Pj23119-sgRNA-PP_3733, homologous arms(two 1.0 kb arms) | This work |
| pSEVA-gR3889T | Derived from pSEVA-gRNA, Pj23119-sgRNA-PP_3889, homologous arms(two 0.5 kb arms) | This work |
| pSEVA-gR3939-3940T | Derived from pSEVA-gRNA, Pj23119-sgRNA-PP_3940, homologous arms(two 0.5 kb arms) | This work |
| pSEVA-gR3947-3948T | Derived from pSEVA-gRNA, Pj23119-sgRNA-PP_3948, homologous arms(two 0.5 kb arms) | This work |
| pSEVA-gR1706T | Derived from pSEVA-gRNA, Pj23119-sgRNA-PP_1706, homologous arms(two 0.5 kb arms) | This work |
| pSEVA-gR3846T | Derived from pSEVA-gRNA, Pj23119-sgRNA-PP_3846, homologous arms(two 0.5 kb arms) | This work |
| pCAS-ZE0 | oriRK2, Tet^R^，Pcas-dCas9,PrhaB-sgRNA | This work |
| PCAS-ZE1 | oriRK2, Tet^R^, Pcas-dCas9,PrhaB-sgRNA-Pj5 | This work |
| PCAS-ZE2 | oriRK2, Tet^R^, Pcas-dCas9,PrhaB-sgRNA-Pj5 | This work |
| PCAS-ZE3 | oriRK2, Tet^R^, Pcas-dCas9,PrhaB-sgRNA-eGFP | This work |
| pSEVA-J5-eGFP | oriPRO1600/ColE1, GmR, Pj5-eGFP | This work |
| pCpf1-RK2K | Derived from pCAS-RK2K, Pcas-Cpf1, PrhaB-crRNA-pRO1600 | This work |
| pSEVA-cR3361T | Derived from pSEVA-gR3361T, Pj23119-crRNA-PP_3361 | This work |
| pSEVA-cR3733T | Derived from pSEVA-gR3733T, Pj23119-crRNA-PP_3733 | This work |
