# Supplementary material for: Genome editing and transcriptional repression in Pseudomonas putida KT2440 via the type II CRISPR system
Source: Microb Cell Fact. 2018 Mar 13;17:41. doi: 10.1186/s12934-018-0887-x (PMC5851096; doi:10.1186/s12934-018-0887-x)
Supplement: Supplementary file 2 — Additional file 2. Key primers used in this study. [file 12934_2018_887_MOESM2_ESM.docx]

**Additional file 2. Key primers used in this study.**

|  |  |
| --- | --- |
| **Primers name** | **Primer sequence (5’ → 3’)** |
| **Sa-RK-1F** | GATACAAGAGCCATAAGAACCCTTTTCCGCTGCATAACCCTG |
| **Sa-RK-2R** | TGCGGAACTGACTAAAGTAGTGGGCCGCCGGCGTTGTGGATA |
| **Sa-BBR1-1F** | GATACAAGAGCCATAAGAACCCTACCGGCGCGGCAGCGTGAC |
| **Sa-BBR1-2R** | TGCGGAACTGACTAAAGTAGTGCGGCCACCGGCTGGCTCGCT |
| **Sa-Z3-F** | TACTTTTCATACTCCCGCCATTCAGAGAAGAAACCAATTGTC |
| **Sa-Z3-R** | GACAATTGGTTTCTTCTCTGAATGGCGGGAGTATGAAAAGTA |
| **QC-1F** | ATCAGAGATTTTGAGACACAATCTAGACATGAGCGGATACAT |
| **QC-1R** | ATGTATCCGCTCATGTCTAGATTGTGTCTCAAAATCTCTGAT |
| **XC-2F** | TATCGAATCTTCCTACAACCTACGACCAGTCTAAAAAGCGCC |
| **XC-2R** | GGTTGTAGGAAGATTCGATAGTTTTAGAGCTAGAAATAGCAA |
| **JH-1F** | AGTGCCACCTGCATCGATTTACGCATCCTCACGATAATATCC |
| **JH-1R** | GGATATTATCGTGAGGATGCGTAAATCGATGCAGGTGGCACT |
| **JH-2F** | CATGGGTATGGACAGTTTTCCCTTTGATATGTAACGGTGAAC |
| **JH-2R** | GTTCACCGTTACATATCAAAGGGAAAACTGTCCATACCCATG |
| **JH-3F** | AACAATACTTAATACTATAGAATGATAACAAAATAAACTACT |
| **JH-3R** | AGTAGTTTATTTTGTTATCATTCTATAGTATTAAGTATTGTT |
| **JS-1F** | CCGGAATTCTTGACAGCTAGCTCAGTC |
| **JS-2R** | CCGAGCTCCTCAAAAAAAGCACCGACTCG |
| **NC-F** | AAAATCGCAATCGTCGGTGCGTTTTAGAGCTAGAAATAGCAA |
| **NC-R** | GCACCGACGATTGCGATTTTGCTAGCATTATACCTAGGACT |
| **NT-1F** | CGCGGATCCGGCAGCTCTACCGCATACACC |
| **NT-ZF** | AGAACAAGAGGAGACATACCCGCACCTTCGTCGCTGGTGGTA |
| **NT-ZR** | TACCACCAGCGACGAAGGTGCGGGTATGTCTCCTCTTGTTCT |
| **NT-2R** | CCCAAGCTTGTCGCTCCAGGTGGCACA |
| **Nic6F** | AGGCAGAAAATCGCAATCGTGTTTTAGAGCTAGAAATAGCAA |
| **Nic6R** | ACGATTGCGATTTTCTGCCTGCTAGCATTATACCTAGGACTG |
| **Nic5F** | CGGAAGATTTTCATCACGTTGTTTTAGAGCTAGAAATAGCAA |
| **Nic5R** | AACGTGATGAAAATCTTCCGGCTAGCATTATACCTAGGACTG |
| **DgRNA-F** | CATTCAGAACTAACTTGTCGGTTTTAGAGCTAGAAATAGCA |
| **DgRNA-R** | CGACAAGTTAGTTCTGAATGGCTAGCATTATACCTAGGACTG |
| **PDF1F** | ATGGATAAGAAATACTCAATAGGCTTAGCTATCGGCACAAATAGCG |
| **PDF1R** | CGCTATTTGTGCCGATAGCTAAGCCTATTGAGTATTTCTTATCCAT |
| **PDF2F** | TTAAGTGATTATGATGTCGATGCCATTGTTCCACAAAGTTTCCTTA |
| **PDF2R** | TAAGGAAACTTTGTGGAACAATGGCATCGACATCATAATCACTTAA |
| **PDFM-F** | TTTTATTTTAGGAGGCAAAAAGTGGATAAGAAATACTCAATA |
| **PDFM-R** | TATTGAGTATTTCTTATCCACTTTTTGCCTCCTAAAATAAAA |
| **CasFM-258-F** | CCTTAATTAAGGAATACTATAGAATGATAACAAAATAA |
| **CasFM-258-R** | CTAGACTAGTCTAGTTACATATCAAAGGGAAAACTG |
| **PS1** | AGGGCGGCGGATTTGTCC |
| **PS2** | GCGGCAACCGAGCGTTC |
| **Ra-JF** | CAAAACTGGCCTTGTGTTAAAAAT |
| **Ra-JR** | CATGATCGCGTACGAAACAGACCG |
| **NCD1F** | GAGCTCGGTACCCGGGGATCCGGCAGCTCTACCGCATACACC |
| **NCD1R** | GGTGTATGCGGTAGAGCTGCCGGATCCCCGGGTACCGAGCTC |
| **NCD2F** | GCTGAAGCGCCGATCTACACTGCAGGCATGCAAGCTTGCGGCCGC |
| **NCD2R** | GCGGCCGCAAGCTTGCATGCCTGCAGTGTAGATCGGCGCTTCAGC |
| **NPAM-F** | GCAATCGTCAGTGCAGGTCTGG |
| **NPAM-R** | CTGACGATTGCGATTTTCTGCCT |
| **D1-JF** | GGCAGCTCTACCGCATACAC |
| **D1-JR** | CGAAGGCTGCGCGCATTT |
| **A20F** | ATGTCTCATAAGATCATTACCGGTGCAGGTCTGGGTGGGG |
| **A20R** | GTAATGATCTTATGAGACATACGATTGCGATTTTCTGCCTACCCGGCAT |
| **Nic-SF** | GGTGACCTAGTGCGCCTGGACTT |
| **Nic-SR** | GCGCACTAGGTCACCTTCGTCGA |
| **NT20-F** | ATGTCTCATAAGATCATTACGTTTTAGAGCTAGAAATAGC |
| **NT20-R** | GTAATGATCTTATGAGACATGCTAGCATTATACCTAGGAC |
| **NT21-F** | GAAAATCGCAATCGTCGGTGCAGGTCTGGGTGGGGCAGCC |
| **NT21-R** | CCGACGATTGCGATTTTCTGCCTACCCGGCATGGGTATGT |
| **ZEJ-15F** | TTATTGACACAGGTGGAAATTACGACCAGTCTAAAAAGC |
| **ZEJ-15R** | ATTTCCACCTGTGTCAATAAGTTTTAGAGCTAGAAATAG |
| **ZJE-30F** | GTGTCAATAACGGTTTTTATTACGACCAGTCTAAAAAGC |
| **ZJE-30R** | ATAAAAACCGTTATTGACACGTTTTAGAGCTAGAAATAG |
| **ZJE-102F** | CCCTAATAGAATGATCGCGCTACGACCAGTCTAAAAAGC |
| **ZJE-102R** | GCGCGATCATTCTATTAGGGGTTTTAGAGCTAGAAATAG |
| **ZE-J5F** | CTCGTACGAAGGTCGATCCATACGACCAGTCTAAAAAG |
| **ZE-J5R** | TGGATCGACCTTCGTACGAGGTTTTAGAGCTAGAAATA |
| **pCf-1F** | ACTTTTTATTTTAGGAGGCAAAAATGTCCATCTACCAAGAGTTTGT |
| **pCf-1R** | ACAAACTCTTGGTAGATGGACATTTTTGCCTCCTAAAATAAAAAGT |
| **pCf-2F** | TTGTCCAGAACCGCAATAACTAAAGTATATTTTAGATGAAGATTAT |
| **pCf-2R** | ATAATCTTCATCTAAAATATACTTTAGTTATTGCGGTTCTGGACAA |
| **pCf-3F** | GCCCTGCGCCTATCTACAACAGTAGAAATTTACGACCAGTCTAAAAAG |
| **pCf-3R** | TGTAGATAGGCGCAGGGCCGCTTCTTTGAGTTTTTTTGAGGAATACG |
| **361-Cpf1F** | TGTTGTAGATGTACACCGATCCGATCAAGTTCGTTTTTTTGAGGAGCTCGG |
| **361-Cpf1R** | GGTGTACATCTACAACAGTAGAAATTGCTAGCATTATACCTAGGAC |
| **5301-Cpf1F** | CTGTTGTAGATCGCGGGTAAACCCGCCGCTACAATTTTTTTGAGGAGCTC |
| **5301-Cpf1R** | CCCGCGATCTACAACAGTAGAAATTGCTAGCATTATACCTAGGAC |
