# Supplementary figures and images for: Genome editing and transcriptional repression in Pseudomonas putida KT2440 via the type II CRISPR system
Source: Microb Cell Fact. 2018 Mar 13;17:41. doi: 10.1186/s12934-018-0887-x (PMC5851096; doi:10.1186/s12934-018-0887-x)

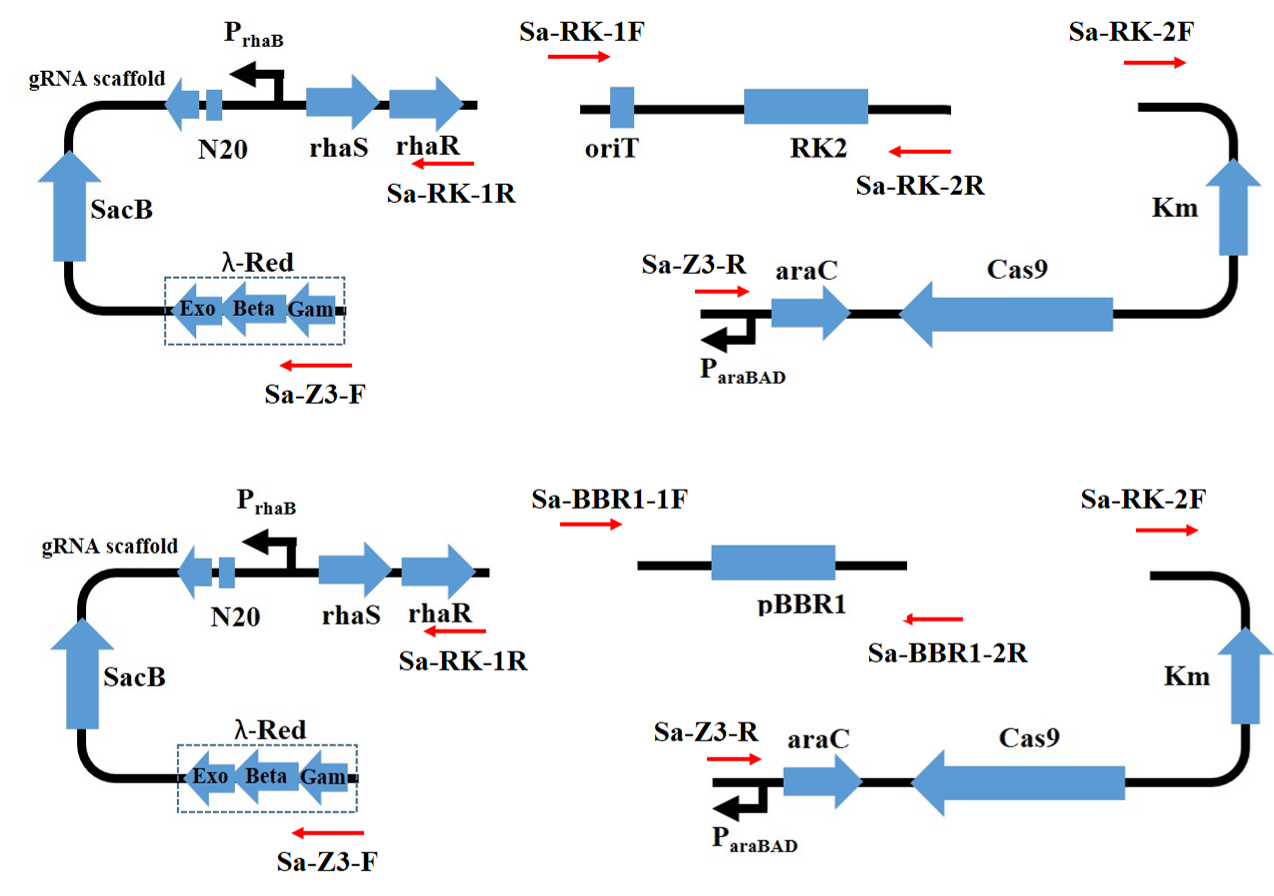

Supplement: Supplementary file 3 — Additional file 3. The construction strategy of pCAS-RK2K and pCAS-pBBR1. [file 12934_2018_887_MOESM3_ESM.bmp]

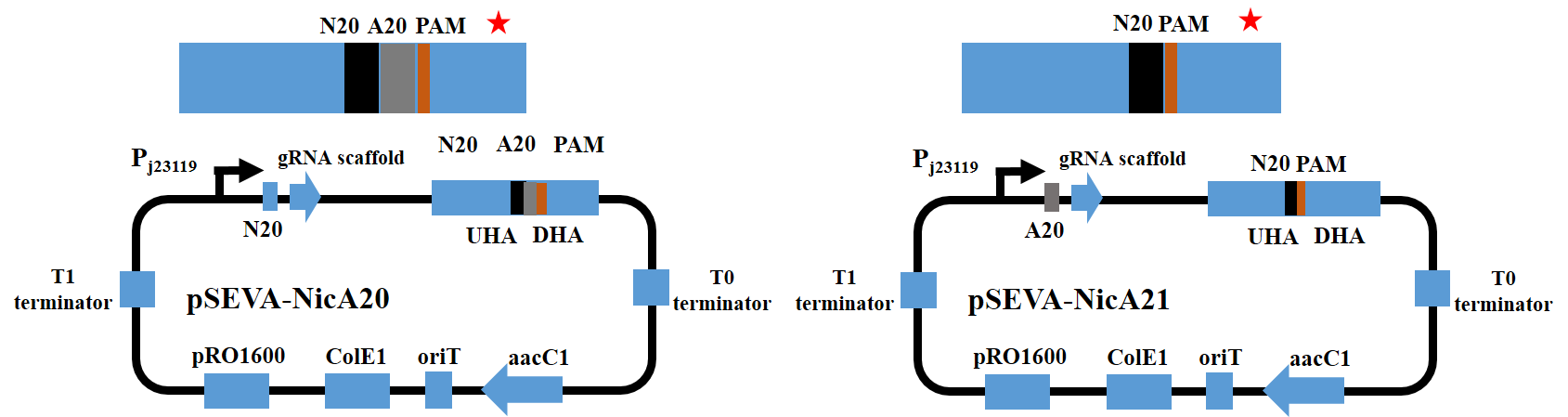

Supplement: Supplementary file 5 — Additional file 5. Plasmids of two-step single nucleotide mutation strategy. [file 12934_2018_887_MOESM5_ESM.bmp]

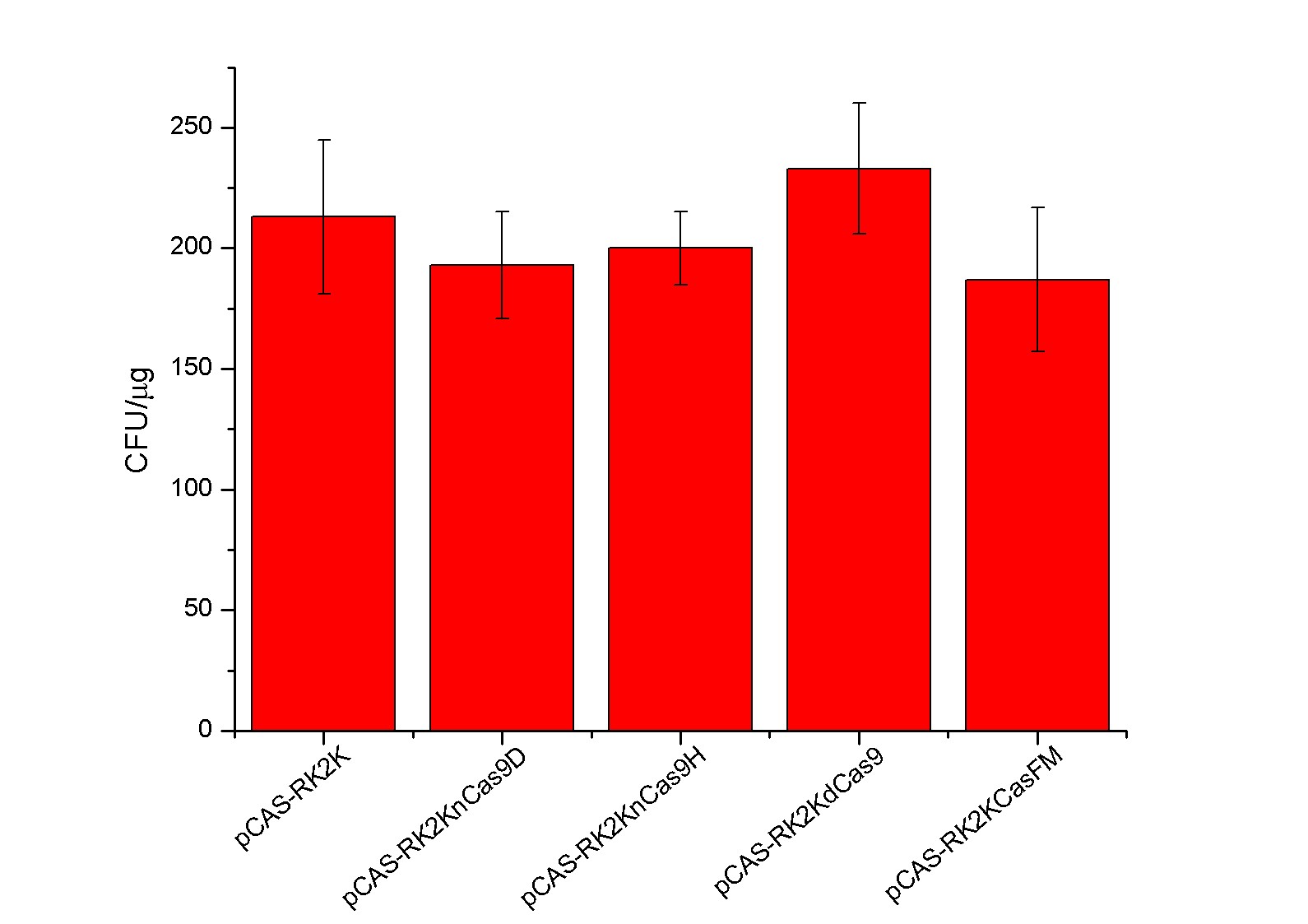

Supplement: Supplementary file 6 — Additional file 6. The total CFU calculation from different Cas9 versions. [file 12934_2018_887_MOESM6_ESM.jpg]

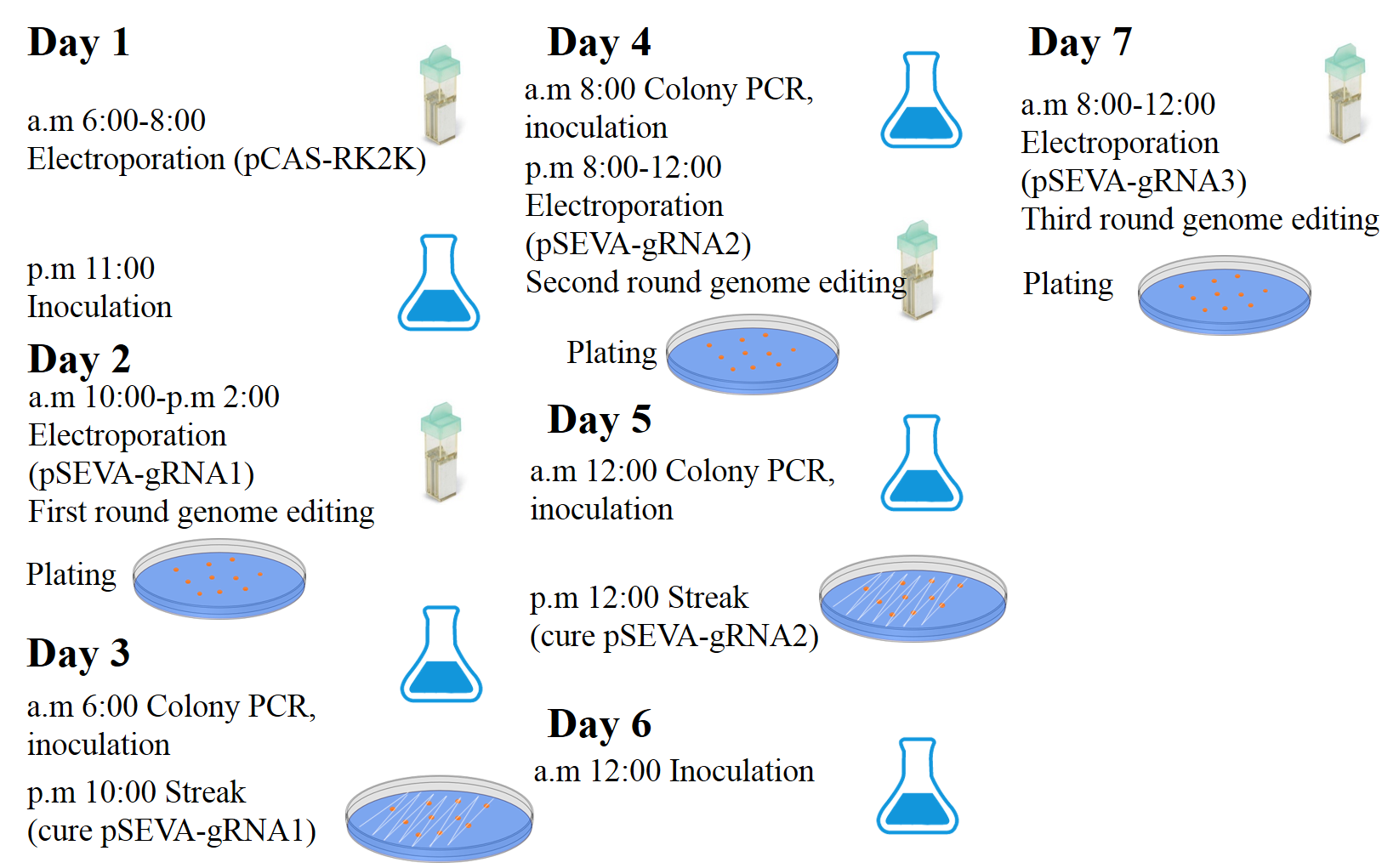

Supplement: Supplementary file 8 — Additional file 8. Flow-chart of 3 rounds of continual genome editing in P. putida KT2440. [file 12934_2018_887_MOESM8_ESM.bmp]
