# Supplementary material for: Genome editing and transcriptional repression in Pseudomonas putida KT2440 via the type II CRISPR system
Source: Microb Cell Fact. 2018 Mar 13;17:41. doi: 10.1186/s12934-018-0887-x (PMC5851096; doi:10.1186/s12934-018-0887-x)
Supplement: Supplementary file 4 — Additional file 4. PAM-guide sequences used in related plasmids. [file 12934_2018_887_MOESM4_ESM.docx]

|  | **N20 sequence designed in Cas9 plasmids** |  |
| --- | --- | --- |
| **Plasmids name** | **N20 sequence** | **DNA Template Strand or target site** |
| **pCAS-RK2K** | **ggttgtaggaagattcgata** | **pRO100** |
| **pCAS-RK2T** | **ggttgtaggaagattcgata** | **pRO100** |
| **pSEVA-gRicT** | **AAAATCGCAATCGTCGGTGC** | **nontemplate strand** |
| **pSEVA-gRicF** | **AAAATCGCAATCGTCGGTGC** | **nontemplate strand** |
| **pSEVA-dgRNA** | **CATTCAGAACTAACTTGTCG** | **No target site** |
| **pSEVA-gRic5T** | **CGGAAGATTTTCATCACGTT** | **template strand** |
| **pSEVA-gRic6F** | **AGGCAGAAAATCGCAATCGT** | **nontemplate strand** |
| **pSEVA-gRic6T** | **AGGCAGAAAATCGCAATCGT** | **nontemplate strand** |
| **pSEVA-gRic6T△NicC::RhaA** | **AGGCAGAAAATCGCAATCGT** | **nontemplate strand** |
| **pSEVA-gRic6T△NicC::dCas9** | **AGGCAGAAAATCGCAATCGT** | **nontemplate strand** |
| **pSEVA-gRic6T△NicC::T7** | **AGGCAGAAAATCGCAATCGT** | **nontemplate strand** |
| **pSEVA-gRic6PAM** | **AGGCAGAAAATCGCAATCGT** | **nontemplate strand** |
| **pSEVA-NicA20** | **AGGCAGAAAATCGCAATCGT** | **nontemplate strand** |
| **pSEVA-NicA21** | **ATGTCTCATAAGATCATTAC** | **A20 sequence** |
| **pSEVA-gR0552T** | **GATGTGAGTTTTGAGTGCAT** | **nontemplate strand** |
| **pSEVA-gR3361T** | **CATTGAAGAAATCTTTTGCA** | **template strand** |
| **pSEVA-gR3733T** | **GGTAGGGCCACAAGGGGACG** | **nontemplate strand** |
| **pSEVA-gR3899T** | **GCGCTGGAGTGAGGTCTCCT** | **nontemplate strand** |
| **pSEVA-gR3939-3940T** | **GGCGTGGGTGTGGATGTTAC** | **nontemplate strand** |
| **pSEVA-gR3947-3948T** | **ACTGTGGATATAGCGCGCAT** | **template strand** |
| **pSEVA-gR3846T** | **TGGCCTGCAATGTACGTTCC** | **nontemplate strand** |
| **pSEVA-gR5301** | **CTCGGTGACGATCTCTTCAG** | **template strand** |
| **pSEVA-gR1706T** | **CAGCCCACGACCGATGATAT** | **template strand** |
| **pSEVA-2gRNA-3733-3361** | **GGTAGGGCCACAAGGGGACG CATTGAAGAAATCTTTTGCA** | **Nontemplate strand template strand** |
| **pCAS-ZE0** | **TGGATCGACCTTCGTACGAG** | **No target site** |
| **PCAS-ZE1** | **ATTTCCACCTGTGTCAATAA** | **J5 promoter** |
| **PCAS-ZE2** | **ATAAAAACCGTTATTGACAC** | **J5 promoter** |
| **PCAS-ZE3** | **GCGCGATCATTCTATTAGGG** | **RBS site of eGFP** |
|  |  |  |
|  | **PAM-guide sequence in Cpf1 plasmids** |  |
| **Plasmids name** | **PAM-guide sequence** | **DNA Template Strand or target site** |
| **pSEVA-cR3361T** | **GTACACCGATCCGATCAAGTTCG** | **template strand** |
| **pSEVA-cR5301T** | **CGCGGGTAAACCCGCCGCTACAA** | **nontemplate strand** |
