# Supplementary material for: Genome editing and transcriptional repression in Pseudomonas putida KT2440 via the type II CRISPR system
Source: Microb Cell Fact. 2018 Mar 13;17:41. doi: 10.1186/s12934-018-0887-x (PMC5851096; doi:10.1186/s12934-018-0887-x)
Supplement: Supplementary file 7 — Additional file 7. The analysis of sgRNA off-target in KT2440 by CasOT. [file 12934_2018_887_MOESM7_ESM.docx]

**Additional file 8. The analysis of sgRNA off-target in KT2440 by CasOT.**

| **pSEVA-gRicT** | **Number of homologous sequence in genome** | **pSEVA-gRic6T** | **Number of homologous sequence in genome** |
| --- | --- | --- | --- |
| A00 | 1 | A00 | 1 |
| A14 | 1 | A23 | 1 |
| A15 | 1 | A24 | 4 |
| A17 | 2 | A25 | 8 |
| A22 | 1 | A26 | 15 |
| A23 | 2 | A27 | 2 |
| A24 | 6 | B24 | 1 |
| A25 | 6 | B25 | 2 |
| A26 | 8 | B26 | 2 |
| A27 | 8 | B27 | 1 |
| B15 | 1 | C24 | 1 |
| B23 | 2 | C26 | 4 |
| B24 | 4 | C27 | 2 |
| B25 | 8 | C28 | 2 |
| B26 | 6 |  |  |
| B27 | 3 |  |  |
| B28 | 3 |  |  |
| C24 | 1 |  |  |
| C25 | 6 |  |  |
| C26 | 7 |  |  |
| C27 | 8 |  |  |
| C28 | 2 |  |  |

ABC means three models of off-target PAM type.

A: -NGG only (default)

B: -NGG and –NAG

C: -NGG,-NAG and -NNGG

A14: 1 means there is 1bp difference in the last 12bp near PAM sequence, 4 means there are 4 bp difference in the whole N20 sequence.

From our experiment, only 1bp difference in the last 12bp near PAM sequence (just like A14) can increase the possibility of sgRNA off-target. Thus, from our research, we recommend the analysis result A23, or A24 as our target site when N20 sequences are analyzed by CasOT.

Reference:

Xiao A, Cheng Z, Kong L, Zhu Z, Lin S, Gao G, et al. CasOT: A genome-wide Cas9/gRNA off-target searching tool. Bioinformatics. 2014;30:1180–2.
